# Red fescue (*Festuca rubra* L.) variety recognition using subset division and neural networks

**DOI:** 10.1007/s12064-026-00480-z

**Published:** 2026-06-09

**Authors:** Laura Slebioda, Bogna Zawieja

**Affiliations:** Department of Mathematical and Statistical Methods, Poznań University of Live Sciences, Wojska Polskiego 28, 60-637 Poznań, Poland

**Keywords:** MLP, Plant breeding, Multi-class classification, Agriculture, Red fescue

## Abstract

The classification of plant varieties is a key task in plant breeding and variety registration. Red fescue (*Festuca rubra* L.), a widely cultivated grass species, includes numerous closely related varieties, making automated classification a challenging multi-class problem. This study aimed to develop and evaluate a multilayer perceptron (MLP) neural network combined with a subset-based decision framework for accurate classification of red fescue varieties and recognition of previously unseen varieties. The study analyzed 76 varieties described by seven morphological features. To address the complexity of the multi-class problem, the dataset was divided into multiple subsets and the effectiveness of different partitioning strategies was evaluated. A confidence-based and majority-based decision rule (majority ratio ≥ 0.9 and mean Softmax confidence ≥ 0.8) was introduced to improve the reliability of final predictions and enable open set recognition. The model was evaluated using accuracy, precision, F1 score, and recall. The most optimal solution was to divide the dataset into 15 subsets, with the first subset containing six varieties and the remaining subsets containing five varieties each. This approach provided the best balance between predictive performance and decision consistency, enabling correct classification of known varieties and stable detection of unknown samples. Combining MLP neural networks with strategic subset division and confidence-driven decision rules offers a robust solution to high-dimensional, multi-class classification challenges in plant variety recognition. The model’s ability to recognize new varieties is crucial for its practical application, ensuring the algorithm’s flexibility. This is particularly useful in agriculture and horticulture, where new varieties are bred over the years.

**Supplementary Information:**

The online version contains supplementary material available at https://doi.org/10.1007/s12064-026-00480-z.

## Introduction

Red fescue (*Festuca rubra L*.) is one of approximately 100 species within the genus *Festuca*, with seven being utilized as turf-type grasses (Torello 1988). It is considered one of the three most important turfgrasses for cool-humid regions and a major component in turfgrass culture worldwide, including Asia, Australia, Africa, Europe, and the Americas. It is valued for its adaptability to shade, lower water usage compared to other turfgrasses, and its ability to thrive in dry and sandy soil conditions with pH levels between 5.5 and 6.5. Red fescue is extensively used for rehabilitating degraded lands, waste dumpsites, and landfills, as well as for the remediation of contaminated soils (Dradrach et al. 2020). The economic significance of red fescue is considerable, as it plays a critical role in maintaining the quality and sustainability of turfgrass systems used in home lawns, parks, municipal areas, cemeteries, roadsides, airfields, golf courses, and other sports turfs (Torello 1988). Its adaptability to various environmental conditions and its role in rehabilitating degraded lands highlight its essential contribution to turfgrass culture and environmental management. Despite its economic and ecological importance, the breeding of red fescue has been hindered by a lack of high-yielding varieties optimized for specific environmental conditions (Baistruk-Hlodan et al. 2023). Climate change and increasing soil and water stresses have exacerbated the demand for resilient turfgrass varieties (Craine et al. 2013). Therefore, the need to create new varieties is justified. Recent studies highlight the challenges of breeding and selecting turfgrass varieties, particularly in adapting to diverse climates and stress conditions (Wang et al. 2024). However, distinguishing new varieties remains an ongoing challenge, as traditional morphological and agronomic methods are often subjective, time-consuming, and insufficient for modern requirements (Baistruk-Hlodan et al. 2023).

In the registration studies of new crop plant varieties, it is essential to demonstrate the distinctness of the new variety separately for all analyzed traits. However, Sharma (2006) shows that techniques such as multivariate analysis are essential for plant breeding and variety testing, including turfgrasses like red fescue. Moreover, during the meetings of the TWC (Technical Working Party on Automation and Computer Programs) of the UPOV (International Union for the Protection of New Varieties of Plants), the topic of multivariate analysis for DUS (Distinctness, Uniformity, and Stability) testing has been frequently discussed (UPOV 2021). This is particularly true for image analysis (e.g., TWC/13/19 (UPOV 1995), TWC/16/10 (Horgan et al. 1998), TWC/19/6 (Roberts et al. 2001), TWC/22/7 (Horgan et al. 2011), TWC/22/8 (Gensollen et al. 2011), TWA/33/7 (UPOV 2015), TWC/24/15 (UPOV 2006), TWC/26/11 (UPOV 2008), TWC/29/19 (Roberts et al. 2011), TWC/29/21 (Markkanen 2011) and spatial analysis (e.g., TWC/18/1 (UPOV 2000), TWC/19/4 (UPOV 2001).

The necessity of applying these methods is outlined in documents TW0/24/12 (UPOV 1991), C/25/10 (UPOV 1991) and TC/27/3 (UPOV 1991). The Working Party extensively discussed whether the phrase “distinguished […] by at least one characteristic” included the application of multivariate analysis. The majority agreed that excluding this method from distinctness testing would disconnect testing authorities from reality. While applying it to predefined or derived characteristics such as shape (measured through length and width) was not expected to cause issues, applying it to all observed characteristics would require further study. The Working Party agreed to continue the discussion based on documents prepared by experts from the United Kingdom and Germany, particularly focusing on varieties like chrysanthemums that are difficult to distinguish without multivariate analysis. In document C/25/10 (UPOV 1991), it was agreed that using multivariate analysis for different parameters of a complex characteristic like shape aligns with the definition of a variety. However, its application to all characteristics, whether related or not, remains unresolved and requires further discussion with input from the Administrative and Legal Committee.

Traditional multivariate approaches often require significant manual intervention and may struggle to handle high-dimensional datasets effectively. Deep learning, specifically Multilayer Perceptron (MLP) models, offers a promising solution. MLPs are a class of feedforward artificial neural networks composed of input, hidden, and output layers, where each neuron is fully connected to the next layer (Gardner and Dorling 1998; Rosenblatt 1958). These models are capable of learning complex nonlinear relationships in multidimensional data, making them suitable for classification tasks involving subtle differences between classes. Unlike traditional statistical methods, MLPs can automatically extract relevant patterns from input features without the need for manual feature engineering (Abiodun et al. 2018). This makes them especially valuable in plant sciences, where high-dimensional phenotypic or spectral data are commonly used. For example, Kamilaris and Prenafeta-Boldú (2018) review the application of deep learning in agriculture, highlighting the use of MLPs in precision agriculture. Although MLPs have been successfully applied to species-level classification problems, their use in turfgrass breeding particularly for the classification of red fescue varieties has been limited. Employing MLPs in this context enables a data-driven and scalable approach to variety recognition, overcoming the limitations of conventional techniques and offering a robust solution for modern plant breeding challenges.

Moreover, handling class imbalance and ensuring reliable decision-making are critical challenges in multi-class classification problems. Class weighting adjusts the contribution of each class to the loss function, allowing the model to place greater emphasis on underrepresented classes during training (King and Zeng 2001; Bakirarar and Elhan 2023). This approach has been shown to be effective in improving classification performance in imbalanced datasets without introducing potential biases associated with data resampling. In addition, confidence-based and majority-based decision rule are incorporated to improve the robustness of classification outcomes. Such approaches are related to the concept of abstaining classification, where predictions are accepted only when sufficient confidence is achieved, reducing the risk of incorrect assignments in uncertain cases (Chow 1970). Confidence-based filtering mechanisms are particularly useful in complex classification tasks, where overlapping class distributions and limited feature representation may lead to ambiguous predictions (El-Yaniv and Wiener 2010).

The aim of this study was to develop a neural network algorithm to distinguish different varieties of red fescue (*Festuca rubra* L.) and demonstrate that subset division is an effective method for handling multi-class classification data. Specifically, for *Festuca rubra* L., dividing the dataset into 15 subsets (first subset containing six varieties and the remaining subsets containing five varieties each) was found to be the most optimal option. The study sought to enhance classification accuracy and provide a practical tool for breeders. Additionally, the algorithm was designed to recognize new varieties, addressing the critical need for adaptability in agricultural and horticultural applications.

## Materials and methods

### Experimental data

The dataset used in this study consisted of numeric data describing morphological characteristics of red fescue (*Festuca rubra* L.) varieties. A total of 131 varieties were initially considered, however, due to significant data gaps – such as missing measurements for key characteristics or incomplete records across multiple blocks – 55 varieties were excluded. These exclusions ensured that the dataset used for analysis was complete and reliable, avoiding potential biases or inaccuracies that could arise from imputed or missing data. Consequently, 76 varieties were ultimately included in the analysis. The selection of varieties was based on the availability of complete measurement data for all defined characteristics across all experimental blocks. Exclusion criteria included varieties with significant data gaps (more than 30% of missing measurements across the characteristics) and outlier varieties with inconsistent growth patterns or abnormalities potentially influenced by experimental errors or environmental anomalies.

The experiments were conducted in a randomized complete block design (using 6 blocks). From each block, 10 plants were randomly selected for observations and measurements. The following morphological characteristics were measured: width of leaves in autumn, natural height of plants in spring, length of the longest stem, length of the upper internode of the stem, length of flag leaf, width of flag leaf, length of inflorescence.

### Model description

In this study, a Multilayer Perceptron (MLP) model was employed for the classification of plant varieties. Multilayer perceptrons (MLPs) are feedforward neural networks composed of several fully connected layers, as established in prior studies (Gardner and Dorling 1998; Rosenblatt 1958). Batch Normalization was applied to improve classification performance and contribute to more stable learning. This layer significantly speeds up neural network training by normalizing the inputs, stabilizing their means and variances (Ioffe and Szegedy 2015). Batch Normalization improves gradient flow through the network by minimizing the sensitivity of gradients to parameter scales and initializations, thereby facilitating more stable and efficient training. Dropout layers were incorporated as a regularization method to reduce the risk of overfitting, a common issue in artificial neural networks. Dropout operates by randomly deactivating neuron units during each iteration of the learning process, thereby reducing interconnections and enhancing generalization (Dewantara et al. 2020). The dense layer is made of fully-connected neurons, meaning each neuron receives input from all neurons in the preceding layer. Algorithm implementation utilizes a sequential model structure.

Table 1 illustrates the sequential model applied to analyze *Festuca rubra* L. varieties. Using the Keras library (a Python-based machine learning framework), the architecture was constructed with five Dense layers, two Batch Normalization layers, and three Dropout layers. Each layer is configured with specific parameters including the number of units and activation function. The activation function in layers 1, 4, 7 and 9 is Rectified Linear Unit (ReLU) which activates a neuron only if the input is positive, helping the model learn complex patterns while mitigating vanishing gradient issues. As noted by Agarap (2018), ReLU introduces non-linearity to the network while maintaining computational efficiency, making it suitable for multi-layer perceptrons. In layer 10 the activation function is Softmax, which as described by Sharma et al. (2020), incorporates multiple sigmoid functions. As sigmoid functions inherently yield values between 0 and 1, they represent the likelihood of data points belonging to particular classes. The Softmax activation function is specifically designed for multi-class classification problems, converting raw outputs into probabilistic distributions across all possible classes.

**Table 1:** Model summary (Model: “sequential”)

Layer Number	Layer	Output shape	Parameter
1	dense	(None, 256)	1792
2	batch_normalization	(None, 256)	1024
3	dropout	(None, 256)	0
4	dense_1	(None, 128)	32,896
5	batch_normalization_1	(None, 128)	512
6	dropout_1	(None, 128)	0
7	dense_2	(None, 64)	8256
8	dropout_2	(None, 64)	0
9	dense_3	(None, 32)	2080
10	dense_4	(None, 76)	2508
	Total parameters:	49,068	
	Trainable parameters:	48,300	
	Non-trainable parameters:	768	

The model employed the categorical cross-entropy loss function, utilized the adam optimizer and measured performance using the accuracy metric. The algorithm was trained for 100 epochs, with a batch size of 40 for processing training data batches. Given the limited number of observations for each variety, class imbalance was addressed by applying class weighting during the training of the MLP model, allowing the network to place greater emphasis on underrepresented classes. The performance of the algorithm was then evaluated using metrics such as accuracy, F1 score, precision and recall. Classification accuracy represents the proportion of correctly predicted instances relative to the total number of cases evaluated (Chicco and Jurman 2020). The precision metric evaluates a model’s exactness by calculating what percentage of its positive classifications were truly correct identifications (Powers 2011). The recall metric quantifies what fraction of actual positive cases were successfully detected by the classifier. F1 score represents precision and recall in one metric. It effectively relates the number of true positives to the arithmetic mean of predicted and actual positives, serving as a normalized metric relative to an ideal value.

The results for known and unknown varieties were also checked, where the known variety was variety 31 and the unknown variety was variety 8094; both were randomly selected. The data used for these tests were not used in creating the algorithm (i.e., the algorithm had never seen them before). The algorithm determines whether a given variety is classified as a new one or matches one of the known varieties, and if so, identifies which variety it is.

The computations were conducted in Python 3.12.2, utilizing libraries such as tensorflow, scikit-learn, pandas, numpy, torch, scipy, matplotlib, and seaborn.

### Application of the model to the data

The dataset was divided into subsets to determine the most optimal division. A total of 7 datasets were created, with information about them presented in Table 2. The created MLP model in the Keras library was applied to each subset.

**Table 2:** Division of the variety dataset into subsets

Set name	Number of subsets	Number of varieties in each subset
DATA	–	76 varieties in one dataset
DATA a	5	16 varieties in subset a1 and 15 in others
DATA b	7	10 varieties in subset b1 and 11 in others
DATA c	10	8 varieties in subsets c1, c2, c3, c4, c5, c6 and 7 in others
DATA d	15	6 varieties in subset d1 and 5 in others
DATA e	19	4 varieties in each subset
DATA f	38	2 varieties in each subset

The input data consisted of seven features describing a *Festuca rubra* L. variety and the labeled output corresponded to an assigned numerical variety identifier. The dataset was divided into training and test sets, with 20% of the data reserved for testing. The split was stratified to preserve class proportions, and a fixed random seed (random_state = 42) was used to ensure reproducibility. The output labels were transformed into binary form through a process known as one-hot encoding. The model was evaluated separately for each subset and in the end, mean was calculated for all evaluation metrics.

The model also considers the possibility of previously unknown varieties. When assigning a given sample to a specific variety, the model may classify it as a novel variety if sufficient consistency and confidence are not achieved. For this purpose, each evaluated sample consisted of 10 observations, both for known and unknown variety. The model systematically evaluates all subsets by assigning each observation to a particular variety and then assesses the consistency of these assignments. For each subset, a majority ratio (i.e., the proportion of observations assigned to the most frequent variety) and the mean prediction confidence are calculated. A subset is considered to represent a known variety only if the majority ratio is at least 0.9 and the mean prediction confidence is at least 0.8. Otherwise, the subset is classified as representing a novel variety. If at least one subset meets these criteria and consistently indicates a specific known variety, the model assigns the entire dataset to that variety. Conversely, if none of the subsets satisfy the required thresholds, the model concludes that the data correspond to a previously unclassified variety.

The operation of partitioning into subsets starts with the user inputting the number of subsets into the variable *n*. Then, the variable *i*, which represents the iteration counter, is initialized to 1. Next, created MLP model is applied. Plots showing training and validation accuracy, training and validation loss, confusion matrix are displayed. Evaluation metrics and classification decision for the new data are also printed. The procedure is repeated until *i* reaches *n*, with the counter incremented after each iteration. Finally, the mean values of the evaluation metrics across all subsets are computed, and the final classification decision is reported.

To additionally assess the effectiveness of the proposed approach, a complementary experiment was performed using a Random Forest (RF) classifier. The model consisted of 100 decision trees, each with a maximum depth of 10 to reduce the risk of overfitting. To encourage tree diversity, only the square root of the total number of features was evaluated at each split. Furthermore, a minimum of five samples was required to perform a node split, and each resulting leaf node was constrained to contain at least two samples. In addition, the same majority-based and confidence-based thresholding rule used in the MLP framework (majority ratio ≥ 0.9 and mean confidence ≥ 0.8) was also applied to the RF predictions to ensure consistency in the decision aggregation process.

## Results

The plots and detailed results for each subset are available in the appendix.

The MLP results obtained from the DATA dataset, detailed in Table 3, were unsatisfactory. This dataset was not divided into subsets, highlighting the difficulty in discriminating among 76 varieties of *Festuca rubra* L. In response, the dataset was partitioned into subsets. Notably, the performance of the algorithm was influenced by the number of subsets, with certain configurations leading to improved evaluation metrics and more stable classification behaviour.

**Table 3:** MLP results for each dataset

Set name	Evaluation metrics				Classification results		
	Accuracy (%)	F1 Score	Precision	Recall	For variety 31		For variety 8094
DATA	12.42	0.100	0.096	0.124	Classified as a new variety.		Classified as a new variety.
DATA a	34.34	0.330	0.340	0.343	Classified as a new variety.		Classified as a new variety.
DATA b	38.35	0.377	0.388	0.384	Classified as a new variety.		Classified as a new variety.
DATA c	47.34	0.468	0.474	0.473	Classified as a new variety.		Classified as a new variety.
DATA d	58.21	0.577	0.588	0.582	Classified as variety: 31.		Classified as a new variety.
DATA e	25.37	0.110	0.090	0.254	Classified as variety: 73 or 114 or 92 or 69 or 103 or 93 or 20 or 31 or 75 or 106 or 33 or 80 or 82 or 8083 or 10 or 49 or 110 or 28.		Classified as variety: 73 or 114 or 88 or 69 or 103 or 93 or 20 or 31 or 106 or 80 or 8083 or 10 or 49 or 110 or 28.
DATA f	80.35	0.802	0.809	0.803	Classified as variety: 88 or 97 or 82 or 58 or 25 or 11 or 20 or 112 or 30 or 80 or 83 or 92 or 3 or 5 or 8 or 42 or 105 or 94 or 59 or 102.		Classified as variety: 108 or 43 or 75 or 112 or 8001 or 72 or 4 or 29 or 76 or 31 or 37 or 46 or 58 or 28 or 98 or 101 or 50.

In terms of the classification outcomes for the selected varieties, a MLP algorithm correctly identified variety 8094 as novel across datasets: DATA, DATA a, DATA b, DATA c and DATA d. Variety 31 was accurately classified solely within dataset DATA d. This demonstrates that dividing the dataset into 15 subsets, with the first subset containing six varieties and the remaining subsets containing five varieties each, was the optimal choice for the created algorithm aimed at distinguishing *Festuca rubra* L. varieties, as it provided the best balance between predictive performance and generalization ability. This setup achieved the highest overall evaluation metrics (58.21% accuracy, 0.577 F1-score, 0.588 precision and 0.582 recall), while also enabling correct identification of variety 31 without misclassifying the known sample as a novel variety. At the same time, it maintained consistent detection of variety 8094 as a new variety across datasets, indicating stable discrimination between known and unknown classes. The selection of the optimal partitioning strategy was based not only on classical evaluation metrics, but also on the consistency of class assignments across subsets. Specifically, a preferable configuration is characterized by: (i) high predictive performance on known varieties, (ii) low ambiguity in subset-level predictions (i.e., a limited number of distinct predicted classes per sample, typically not exceeding two dominant classes), and (iii) stable recognition of unknown samples as novel varieties across all subsets. Configurations producing a large number of conflicting class assignments were considered less reliable due to increased classification uncertainty and reduced interpretability.

Conversely, dataset DATA f, with each subset containing two varieties, exhibited the poorest performance. Despite achieving very high accuracy and other evaluation metrics, the algorithm misclassified 20 distinct varieties as variety 31 and what is worth noting, excluded variety 31 itself from this subset. In dataset DATA e, an additional effect was observed where the model frequently assigned the same predicted class to both the known variety and the unknown variety across multiple subsets, despite relatively low overall accuracy.

A summary analysis of the confusion matrices revealed that misclassification patterns were not random, but consistently occurred between specific pairs of varieties across different dataset configurations. For example, confusion between varieties 83 and 114 was observed in both DATA a and DATA c, while misclassification between varieties 4 and 2 appeared in DATA c and DATA d. This indicates that certain varieties exhibit high similarity in the considered feature space, leading to systematic classification ambiguity.

The results of the supplementary experiment employing a RF classifier are summarized in Table 4. This model demonstrated similar evaluation metrics compared to MLP but was unable to correctly classify variety 31. A contrasting behaviour was observed between the MLP and Random Forest models in dataset DATA e. Both models exhibited different types of limitations under the same threshold-based decision framework. While the MLP frequently assigned the same class to both the known and unknown varieties, indicating a collapse of predictions due to limited discriminative power, Random Forest model correctly identified the unknown variety as a new class, indicating better separation between known and unknown samples. However, for the known variety, it produced multiple competing class assignments (93, 20, 49) and excluded variety 31.

**Table 4:** Random forest results for each dataset

Set name	Evaluation metrics				Classification results		
	Accuracy (%)	F1 Score	Precision	Recall	For variety 31		For variety 8094
DATA	12.54	0.109	0.115	0.125	Classified as a new variety.		Classified as a new variety.
DATA a	33.55	0.325	0.327	0.336	Classified as a new variety.		Classified as a new variety.
DATA b	40.67	0.399	0.402	0.407	Classified as a new variety.		Classified as a new variety.
DATA c	47.22	0.468	0.472	0.472	Classified as a new variety.		Classified as a new variety.
DATA d	59.80	0.595	0.601	0.598	Classified as a new variety.		Classified as a new variety.
DATA e	64.43	0.641	0.647	0.644	Classified as variety: 93 or 20 or 49.		Classified as a new variety.
DATA f	80.73	0.806	0.814	0.807	Classified as variety: 80 or 105 or 5 or 30 or 42 or 25 or 88 or 92 or 94 or 59 or 20 or 82 or 112 or 58 or 3 or 83.		Classified as variety: 76 or 108 or 92 or 70 or 98 or 50 or 28 or 58.

## Discussion

Recognizing different varieties of red fescue using machine learning or deep learning techniques is a niche area of research and there are no studies explicitly focused on this topic. Additionally, it should be noted that many studies concentrate on distinguishing species, and importantly, distinguishing varieties is significantly more challenging than distinguishing species. Furthermore, in studies concerning variety recognition, the number of varieties used to created a model is typically fewer than 76 and is often accompanied by a substantially higher number of input features than 7. Chen et al. (2010) developed a model capable of accurately classifying five corn varieties with 100% accuracy. In their study, a total of 17 geometric features, 13 shape and 28 color features from color images of corn kernels were used. Azizi et al. (2015) achieved 100% accuracy in distinguishing ten potato varieties using neural networks. Researchers selected 16 principal features for this task. In another study, a convolutional neural network was developed that could distinguish eleven barley varieties with 93% accuracy (Kozłowski et al. 2019).

As mentioned earlier, it is much easier to create an algorithm that accurately classifies species. Toğaçar et al. (2020) examined five flower species and created a neural network model that achieved 98.91% accuracy. Quoc Bao et al. (2019) used two datasets, one contained leaf scans of 15 Swedish tree species and the other contained leaf images of 32 different species. The researches developed and compared two models – convolutional neural networks (CNN) and histogram of oriented gradients (HOG). For the first dataset the authors achieved 98.22% accuracy for CNN and 98% accuracy for HOG. For the second dataset there was 95.50% accuracy for CNN and 92% accuracy for HOG.

In our MLP model we can notice low values of evaluation metrics and issues with accurate classification for DATA, DATA a, DATA b, DATA c and DATA e. This indicates the presence of underfitting which is the failure to achieve sufficiently low error rates on training data (Goodfellow et al. 2016). In addition, for DATA e, the model exhibited a tendency to assign the same predicted class to both known and unknown varieties across multiple subsets. This behaviour suggests limited discriminative ability of the model in the given feature space, leading to a bias towards specific classes. Such effects may arise when different classes occupy overlapping regions in the feature space, making it difficult to define clear decision boundaries and increasing the likelihood of misclassification (Xiong et al. 2010). Conversely, despite achieving high values of evaluation metrics for DATA f, the classification was incorrect. Dataset is complex and DATA f has two varieties in each subset. Red fescue varieties may have subtle differences that are difficult for the model to distinguish, even with a sophisticated architecture. With only seven features, it is entirely plausible that the model’s difficulty in distinguishing could be due to the limited variability within each subset. When training data fails to capture the full spectrum of population diversity, machine learning models may amplify existing biases, which can result in lack of generalization (Norori et al. 2021). When each subset contains only a small number of varieties and relatively a small number of features, the model may not encounter enough diversity in the training data to effectively learn the subtle differences between varieties. This lack of variability can hinder the model’s ability to generalize to unseen data, resulting in misclassifications despite high evaluation metrics.

DATA d yielded the most favorable outcomes, with relatively high evaluation metrics and correct classification of variety 31 and variety 8094. Although those metrics may appear moderate compared to conventional classification tasks, they should be interpreted in the context of the exceptionally high complexity of the present problem. The proposed framework attempts to distinguish among 76 closely related red fescue varieties using only seven quantitative morphological features. In variety-level classification tasks, where inter-class differences are often subtle and overlapping, achieving stable discrimination between known and unknown classes represents a substantial challenge. Therefore, the obtained results demonstrate that the proposed subset-based framework is capable of capturing meaningful discriminatory patterns.

The partitioning of the dataset into subsets significantly improved the algorithm’s performance across various evaluation metrics. This division strategy allowed the model to focus more precisely on distinguishing among different varieties of *Festuca rubra* L., thereby enhancing its classification accuracy. Compared to datasets with fewer varieties per subset (like DATA e or DATA f), DATA d contains six varieties in one subset and five varieties in each of the other subsets and likely provide a richer diversity of characteristics. This diversity allows the model to learn more nuanced features that distinguish between different varieties of red fescue. The division into subsets is a well-known method for supporting classification in machine learning but it has never been used in the manner described in this study. Abuassba et al. (2017) utilized subset division to improve classification performance. They divided the dataset into subsets and applied a different classification method to each of the subsets to determine which method yielded the best results.

The optimal number of varieties in a set always varies and different models will yield specific results depending on the number of varieties in a dataset and the number of input features. In a study concerning corn, the dataset contained six varieties and the applied CNN model achieved 99.20% accuracy (Yang et al. 2023). In another study by Chen et al. (2010), also focusing on corn, there were five varieties in the dataset and the model achieved 100% accuracy. This demonstrates that for corn, including five or six varieties in a single dataset appears to be the most optimal option. However, it should also be noted that a lot depends on the developed classification algorithm. In the presented study on *Festuca rubra* L., for the created algorithm, the most optimal option was to include five varieties in a set. With five varieties per subset, the dataset maintains a balance between size and complexity. It provides sufficient variability and examples for the model to generalize well to unseen data while avoiding the pitfalls of overfitting or underfitting. This optimal subset size ensures that the model learns robust representations of each variety. The significant effect of different dataset sizes was confirmed by AbdulAzeem et al. (2021). In the first experiment, for different dataset sizes, the accuracies ranged from 99.95% to 100%. In the second experiment, accuracies ranged from 95.96% to 98.26%.

In addition to classical evaluation metrics, the final decision in the proposed approach was also governed by a confidence-based thresholding mechanism. The classification outcome for each subset was accepted only when two conditions were simultaneously satisfied: (i) a high majority agreement among subset predictions (majority ratio ≥ 0.8) and (ii) a sufficiently high average prediction confidence (mean confidence ≥ 0.9). If these conditions were not met, the sample was treated as an ambiguous case and classified as a new variety. This strategy allowed the model to explicitly distinguish between reliable class assignments and uncertain predictions, thereby improving robustness in identifying previously unseen varieties.

Despite broadly comparable overall performance metrics between the two models, their behaviour within the proposed subset-based classification framework differs substantially. Both the MLP and Random Forest classifiers achieve similar ranges of accuracy, precision, F1-score and recall across datasets; however, these aggregated metrics do not fully capture differences in decision stability and class assignment consistency. The MLP model, as a nonlinear function approximator capable of learning complex feature interactions (Goodfellow et al. 2016; Chollet 2018), combined with a confidence-based and majority-based thresholding mechanism, produces more consolidated classification outcomes across subsets. This is particularly evident in dataset DATA d, where it consistently identifies variety 31 as a stable class while simultaneously maintaining the correct classification of variety 8094 as a novel class. The applied decision rule (majority ratio ≥ 0.8 and mean confidence ≥ 0.9) enforces a reject option for uncertain cases. This strategy reflects the classical error-reject trade-off, where increasing the confidence threshold reduces misclassification at the cost of rejecting more uncertain samples (Chow 1970). In contrast, the Random Forest classifier, despite achieving higher classification accuracy in certain configurations (e.g., DATA e), exhibited greater variability in subset-level class assignments, leading to fragmented decision patterns and reduced consistency in aggregated results. As a consequence, its predictions often failed to meet the proposed aggregation criteria based on majority agreement and confidence thresholds. In comparison, the MLP model, although characterized by lower overall accuracy, produced more stable and consistent predictions across subsets, which enabled correct final classification within the proposed decision framework. These findings indicate that high classification accuracy alone does not guarantee reliable outcomes in a subset-based approach, where consistency of predictions across subsets plays a crucial role in determining the final decision. This observation is consistent with previous findings that classification accuracy alone may not fully reflect the reliability of model decisions (Foody 2023). Overall, model suitability in the proposed multi-subset framework should be evaluated not only in terms of classical performance metrics, but also with respect to prediction stability and compatibility with the applied confidence-based and majority-based decision mechanism.

The model’s ability to correctly classify variety 8094 as a new variety across multiple datasets demonstrates its robustness in identifying previously unseen varieties. This capability underscores the potential of the developed algorithm for practical applications in agriculture. Developing an algorithm that is able to classify data as new, previously unseen variety is not a common approach. Scheirer et al. (2013) named it as open set recognition, where the training set lacks complete class information, requiring algorithms to handle previously unseen categories during testing. Open set recognition is used in agriculture and horticulture mostly for diseases recognition. Fuentes et al. (2021) applied deep learning techniques for tomato disease recognition for the open set and later, Fuentes et al. (2023) used similar methods for plant disease recognition across multiple greenhouse scenarios which was also an open set approach. Meng et al. (2023) not only examined plant diseases recognition but also species recognition for the open set obtaining high accuracy in recognizing known classes and exhibited a robust ability to detect and classify unknown classes as distinct from the known ones.

The presented research shows that using neural networks combined with subset division can effectively improve the classification accuracy of complex datasets. Future studies can explore the application of similar techniques to other plant species or classification problems in different domains. The method of subset division is particularly promising for scenarios with numerous classes and limited data, making it a valuable approach for various applications in machine learning and data analysis. Despite achieving high accuracy in some datasets, challenges such as misclassifications of specific varieties highlight the complexity of distinguishing closely related varieties. Future improvements could focus on refining the model’s sensitivity to such distinctions. Additionally, the composition of subsets may influence the stability of the final classification outcome, suggesting that alternative partitioning strategies should be systematically investigated in future work. Future work will also include repeated runs and cross-validation to assess variability and improve statistical robustness of the results. Importantly, the observed optimal configuration appears to be purely empirical and dataset-dependent and may vary with the structure of the data and the feature space. What is worth noting, the model’s ability to recognize unknown classes is crucial for its practical application, as it ensures robustness in real-world scenarios where new varieties may emerge, thereby enhancing the reliability and adaptability of the classification system. Open set recognition is specifically useful in agriculture and horticulture where new varieties are bred throughout the years.

## Supplementary Information

Below is the link to the electronic supplementary material.

Supplementary Material 1

Supplementary Material 2

Supplementary Material 3

## Data Availability

No datasets were generated or analysed during the current study.
