# Supplementary material for: Red fescue (Festuca rubra L.) variety recognition using subset division and neural networks
Source: Theory Biosci. 2026 Jun 9;145(3):28. doi: 10.1007/s12064-026-00480-z (PMC13246904; doi:10.1007/s12064-026-00480-z)
Supplement: Supplementary file 1 — Supplementary Material 1 [file 12064_2026_480_MOESM1_ESM.docx]

**Article title: Red Fescue (*Festuca rubra* L*.*) Variety Recognition Using Subset Division and Neural Networ****ks**

**Journal name: Theory in Biosciences**

**Author names: Laura Slebioda, Bogna Zawieja**

**Affiliation and e-mail address of the corresponding author: Department of Mathematical and Statistical Methods, Poznań University of Live Sciences, Wojska Polskiego 28, 60-637 Poznań, Poland; laura.slebioda@up.poznan.pl**

**Table 1. Division of varieties into subsets.**

| **Subset** | **Variety** |
| --- | --- |
| DATA a1 | 2, 3, 4, 20, 21, 46, 33, 34, 115, 92, 101, 102, 112, 70, 71, 80 |
| DATA a2 | 30, 31, 49, 50, 56, 64, 65, 75, 76, 81, 97, 106, 107, 8001, 8083 |
| DATA a3 | 5, 6, 22, 23, 37, 85, 96, 98, 99, 100, 108, 110, 111, 8002, 8003 |
| DATA a4 | 8, 10, 11, 25, 28, 29, 42, 43, 54, 66, 69, 77, 79, 88, 89 |
| DATA a5 | 58, 59, 61, 72, 73, 74, 82, 83, 84, 93, 94, 103, 104, 105, 114 |
| DATA b1 | 2, 3, 4, 20, 21, 46, 33, 34, 115, 92 |
| DATA b2 | 30, 31, 81, 8001, 8083, 56, 97, 106, 107, 65, 75 |
| DATA b3 | 77, 79, 88, 66, 69, 89, 42, 43, 54, 25, 28 |
| DATA b4 | 101, 102, 112, 70, 71, 80, 76, 49, 50, 64, 11 |
| DATA b5 | 23, 37, 96, 5, 6, 22, 8002, 8003, 85, 108, 110 |
| DATA b6 | 103, 104, 105, 93, 94, 114, 82, 83, 84, 72, 73 |
| DATA b7 | 29, 8, 10, 111, 98, 99, 100, 74, 58, 59, 61 |
| DATA c1 | 103, 104, 105, 93, 94, 114, 82, 83 |
| DATA c2 | 77, 79, 88, 66, 69, 89, 42, 43 |
| DATA c3 | 23, 37, 96, 5, 6, 22, 8002, 8003 |
| DATA c4 | 30, 31, 81, 8001, 8083, 56, 97, 106 |
| DATA c5 | 2, 3, 4, 20, 21, 46, 33, 34 |
| DATA c6 | 115, 92, 101, 102, 112, 70, 71, 80 |
| DATA c7 | 107, 65, 75, 76, 49, 50, 64 |
| DATA c8 | 85, 108, 110, 111, 98, 99, 100 |
| DATA c9 | 54, 25, 28, 29, 8, 10, 11 |
| DATA c10 | 84, 72, 73, 74, 58, 59, 61 |
| DATA d1 | 103, 104, 105, 93, 94, 114 |
| DATA d2 | 77, 79, 88, 66, 69 |
| DATA d3 | 82, 83, 89, 42, 43 |
| DATA d4 | 23, 37, 96, 5, 6 |
| DATA d5 | 54, 25, 28, 29, 8 |
| DATA d6 | 22, 8002, 8003, 10, 11 |
| DATA d7 | 30, 31, 81, 8001, 8083 |
| DATA d8 | 85, 108, 110, 111, 98 |
| DATA d9 | 56, 97, 106, 99, 100 |
| DATA d10 | 2, 3, 4, 20, 21 |
| DATA d11 | 46, 33, 34, 59, 61 |
| DATA d12 | 84, 72, 73, 74, 58 |
| DATA d13 | 115, 92, 101, 102, 112 |
| DATA d14 | 107, 65, 75, 76, 49 |
| DATA d15 | 70, 71, 80, 50, 64 |
| DATA e1 | 104, 105, 93, 115 |
| DATA e2 | 23, 37, 96, 103 |
| DATA e3 | 84, 72, 73, 74 |
| DATA e4 | 94, 114, 6, 58 |
| DATA e5 | 92, 101, 102, 5 |
| DATA e6 | 107, 65, 75, 76 |
| DATA e7 | 46, 33, 34, 59 |
| DATA e8 | 2, 3, 4, 20 |
| DATA e9 | 112, 49, 61, 21 |
| DATA e10 | 56, 97, 106, 99 |
| DATA e12 | 85, 108, 110, 111 |
| DATA e13 | 30, 31, 81, 8001 |
| DATA e14 | 100, 98, 8083, 11 |
| DATA e15 | 54, 25, 28, 29 |
| DATA e16 | 82, 83, 89, 42 |
| DATA e17 | 70, 71, 80, 50 |
| DATA e18 | 77, 79, 88, 66 |
| DATA e19 | 8, 43, 64, 69 |
| DATA f | In each subset are 2 varieties, in ascending order. |

**Table 2. MLP Results.**

| Set name | DATA |
| --- | --- |
| Classification results for variety 31 | The new data is classified as a new variety. |
| Classification results for variety 8094 | The new data is classified as a new variety. |
| Accuracy | 0.12 |
| F1 Score | 0.10 |
| Precision | 0.10 |
| Recall | 0.12 |

| Set name | DATA a |
| --- | --- |
| Subset name | DATA a1 |
| Mean confidence for variety 31 data | 0.37 |
| Majority ratio for variety 31 data | 0.30 |
| Classification results for variety 31 | The new data is classified as a new variety. |
| Mean confidence for variety 8094 data | 0.48 |
| Majority ratio for variety 8094 data | 0.60 |
| Classification results for variety 8094 | The new data is classified as a new variety. |
| Accuracy | 0.36 |
| F1 Score | 0.34 |
| Precision | 0.36 |
| Recall | 0.36 |

| Set name | DATA a |
| --- | --- |
| Subset name | DATA a2 |
| Mean confidence for variety 31 data | 0.31 |
| Majority ratio for variety 31 data | 0.50 |
| Classification results for variety 31 | The new data is classified as a new variety. |
| Mean confidence for variety 8094 data | 0.39 |
| Majority ratio for variety 8094 data | 0.30 |
| Classification results for variety 8094 | The new data is classified as a new variety. |
| Accuracy | 0.35 |
| F1 Score | 0.33 |
| Precision | 0.34 |
| Recall | 0.35 |

| Set name | DATA a |
| --- | --- |
| Subset name | DATA a3 |
| Mean confidence for variety 31 data | 0.33 |
| Majority ratio for variety 31 data | 0.40 |
| Classification results for variety 31 | The new data is classified as a new variety. |
| Mean confidence for variety 8094 data | 0.58 |
| Majority ratio for variety 8094 data | 0.40 |
| Classification results for variety 8094 | The new data is classified as a new variety. |
| Accuracy | 0.40 |
| F1 Score | 0.38 |
| Precision | 0.39 |
| Recall | 0.40 |

| Set name | DATA a |
| --- | --- |
| Subset name | DATA a4 |
| Mean confidence for variety 31 data | 0.28 |
| Majority ratio for variety 31 data | 0.4 |
| Classification results for variety 31 | The new data is classified as a new variety. |
| Mean confidence for variety 8094 data | 0.40 |
| Majority ratio for variety 8094 data | 0.60 |
| Classification results for variety 8094 | The new data is classified as a new variety. |
| Accuracy | 0.32 |
| F1 Score | 0.31 |
| Precision | 0.32 |
| Recall | 0.32 |

| Set name | DATA a |
| --- | --- |
| Subset name | DATA a5 |
| Mean confidence for variety 31 data | 0.41 |
| Majority ratio for variety 31 data | 0.50 |
| Classification results for variety 31 | The new data is classified as a new variety. |
| Mean confidence for variety 8094 data | 0.37 |
| Majority ratio for variety 8094 data | 0.30 |
| Classification results for variety 8094 | The new data is classified as a new variety. |
| Accuracy | 0.29 |
| F1 Score | 0.28 |
| Precision | 0.29 |
| Recall | 0.29 |

| Set name | DATA b |
| --- | --- |
| Subset name | DATA b1 |
| Mean confidence for variety 31 data | 0.47 |
| Majority ratio for variety 31 data | 0.60 |
| Classification results for variety 31 | The new data is classified as a new variety. |
| Mean confidence for variety 8094 data | 0.63 |
| Majority ratio for variety 8094 data | 0.3 |
| Classification results for variety 8094 | The new data is classified as a new variety. |
| Accuracy | 0.41 |
| F1 Score | 0.41 |
| Precision | 0.43 |
| Recall | 0.41 |

| Set name | DATA b |
| --- | --- |
| Subset name | DATA b2 |
| Mean confidence for variety 31 data | 0.41 |
| Majority ratio for variety 31 data | 0.80 |
| Classification results for variety 31 | The new data is classified as a new variety. |
| Mean confidence for variety 8094 data | 0.59 |
| Majority ratio for variety 8094 data | 0.30 |
| Classification results for variety 8094 | The new data is classified as a new variety. |
| Accuracy | 0.40 |
| F1 Score | 0.39 |
| Precision | 0.40 |
| Recall | 0.40 |

| Set name | DATA b |
| --- | --- |
| Subset name | DATA b3 |
| Mean confidence for variety 31 data | 0.41 |
| Majority ratio for variety 31 data | 0.40 |
| Classification results for variety 31 | The new data is classified as a new variety. |
| Mean confidence for variety 8094 data | 0.44 |
| Majority ratio for variety 8094 data | 0.40 |
| Classification results for variety 8094 | The new data is classified as a new variety. |
| Accuracy | 0.30 |
| F1 Score | 0.29 |
| Precision | 0.29 |
| Recall | 0.30 |

| Set name | DATA b |
| --- | --- |
| Subset name | DATA b4 |
| Mean confidence for variety 31 data | 0.40 |
| Majority ratio for variety 31 data | 0.40 |
| Classification results for variety 31 | The new data is classified as a new variety. |
| Mean confidence for variety 8094 data | 0.48 |
| Majority ratio for variety 8094 data | 0.40 |
| Classification results for variety 8094 | The new data is classified as a new variety. |
| Accuracy | 0.35 |
| F1 Score | 0.35 |
| Precision | 0.35 |
| Recall | 0.35 |

| Set name | DATA b |
| --- | --- |
| Subset name | DATA b5 |
| Mean confidence for variety 31 data | 0.41 |
| Majority ratio for variety 31 data | 0.30 |
| Classification results for variety 31 | The new data is classified as a new variety. |
| Mean confidence for variety 8094 data | 0.60 |
| Majority ratio for variety 8094 data | 0.50 |
| Classification results for variety 8094 | The new data is classified as a new variety. |
| Accuracy | 0.46 |
| F1 Score | 0.45 |
| Precision | 0.47 |
| Recall | 0.46 |

| Set name | DATA b |
| --- | --- |
| Subset name | DATA b6 |
| Mean confidence for variety 31 data | 0.32 |
| Majority ratio for variety 31 data | 0.80 |
| Classification results for variety 31 | The new data is classified as a new variety. |
| Mean confidence for variety 8094 data | 0.38 |
| Majority ratio for variety 8094 data | 0.20 |
| Classification results for variety 8094 | The new data is classified as a new variety. |
| Accuracy | 0.36 |
| F1 Score | 0.35 |
| Precision | 0.37 |
| Recall | 0.36 |

| Set name | DATA b |
| --- | --- |
| Subset name | DATA b7 |
| Mean confidence for variety 31 data | 0.41 |
| Majority ratio for variety 31 data | 0.50 |
| Classification results for variety 31 | The new data is classified as a new variety. |
| Mean confidence for variety 8094 data | 0.46 |
| Majority ratio for variety 8094 data | 0.50 |
| Classification results for variety 8094 | The new data is classified as a new variety. |
| Accuracy | 0.40 |
| F1 Score | 0.39 |
| Precision | 0.42 |
| Recall | 0.40 |

| Set name | DATA c |
| --- | --- |
| Subset name | DATA c1 |
| Mean confidence for variety 31 data | 0.58 |
| Majority ratio for variety 31 data | 0.60 |
| Classification results for variety 31 | The new data is classified as a new variety. |
| Mean confidence for variety 8094 data | 0.63 |
| Majority ratio for variety 8094 data | 0.40 |
| Classification results for variety 8094 | The new data is classified as a new variety. |
| Accuracy | 0.43 |
| F1 Score | 0.41 |
| Precision | 0.42 |
| Recall | 0.43 |

| Set name | DATA c |
| --- | --- |
| Subset name | DATA c2 |
| Mean confidence for variety 31 data | 0.36 |
| Majority ratio for variety 31 data | 0.40 |
| Classification results for variety 31 | The new data is classified as a new variety. |
| Mean confidence for variety 8094 data | 0.51 |
| Majority ratio for variety 8094 data | 0.30 |
| Classification results for variety 8094 | The new data is classified as a new variety. |
| Accuracy | 0.45 |
| F1 Score | 0.45 |
| Precision | 0.45 |
| Recall | 0.45 |

| Set name | DATA c |
| --- | --- |
| Subset name | DATA c3 |
| Mean confidence for variety 31 data | 0.47 |
| Majority ratio for variety 31 data | 0.30 |
| Classification results for variety 31 | The new data is classified as a new variety. |
| Mean confidence for variety 8094 data | 0.80 |
| Majority ratio for variety 8094 data | 0.40 |
| Classification results for variety 8094 | The new data is classified as a new variety. |
| Accuracy | 0.53 |
| F1 Score | 0.54 |
| Precision | 0.55 |
| Recall | 0.53 |

| Set name | DATA c |
| --- | --- |
| Subset name | DATA c4 |
| Mean confidence for variety 31 data | 0.56 |
| Majority ratio for variety 31 data | 0.90 |
| Classification results for variety 31 | The new data is classified as a new variety. |
| Mean confidence for variety 8094 data | 0.59 |
| Majority ratio for variety 8094 data | 0.90 |
| Classification results for variety 8094 | The new data is classified as a new variety. |
| Accuracy | 0.50 |
| F1 Score | 0.50 |
| Precision | 0.51 |
| Recall | 0.50 |

| Set name | DATA c |
| --- | --- |
| Subset name | DATA c5 |
| Mean confidence for variety 31 data | 0.52 |
| Majority ratio for variety 31 data | 0.50 |
| Classification results for variety 31 | The new data is classified as a new variety. |
| Mean confidence for variety 8094 data | 0.55 |
| Majority ratio for variety 8094 data | 0.30 |
| Classification results for variety 8094 | The new data is classified as a new variety. |
| Accuracy | 0.50 |
| F1 Score | 0.49 |
| Precision | 0.50 |
| Recall | 0.50 |

| Set name | DATA c |
| --- | --- |
| Subset name | DATA c6 |
| Mean confidence for variety 31 data | 0.54 |
| Majority ratio for variety 31 data | 0.50 |
| Classification results for variety 31 | The new data is classified as a new variety. |
| Mean confidence for variety 8094 data | 0.57 |
| Majority ratio for variety 8094 data | 0.50 |
| Classification results for variety 8094 | The new data is classified as a new variety. |
| Accuracy | 0.56 |
| F1 Score | 0.55 |
| Precision | 0.56 |
| Recall | 0.56 |

| Set name | DATA c |
| --- | --- |
| Subset name | DATA c7 |
| Mean confidence for variety 31 data | 0.42 |
| Majority ratio for variety 31 data | 0.30 |
| Classification results for variety 31 | The new data is classified as a new variety. |
| Mean confidence for variety 8094 data | 0.55 |
| Majority ratio for variety 8094 data | 0.50 |
| Classification results for variety 8094 | The new data is classified as a new variety. |
| Accuracy | 0.40 |
| F1 Score | 0.39 |
| Precision | 0.39 |
| Recall | 0.40 |

| Set name | DATA c |
| --- | --- |
| Subset name | DATA c8 |
| Mean confidence for variety 31 data | 0.59 |
| Majority ratio for variety 31 data | 0.60 |
| Classification results for variety 31 | The new data is classified as a new variety. |
| Mean confidence for variety 8094 data | 0.63 |
| Majority ratio for variety 8094 data | 0.50 |
| Classification results for variety 8094 | The new data is classified as a new variety. |
| Accuracy | 0.50 |
| F1 Score | 0.50 |
| Precision | 0.51 |
| Recall | 0.50 |

| Set name | DATA c |
| --- | --- |
| Subset name | DATA c9 |
| Mean confidence for variety 31 data | 0.39 |
| Majority ratio for variety 31 data | 0.40 |
| Classification results for variety 31 | The new data is classified as a new variety. |
| Mean confidence for variety 8094 data | 0.50 |
| Majority ratio for variety 8094 data | 0.30 |
| Classification results for variety 8094 | The new data is classified as a new variety. |
| Accuracy | 0.46 |
| F1 Score | 0.46 |
| Precision | 0.46 |
| Recall | 0.46 |

| Set name | DATA c |
| --- | --- |
| Subset name | DATA c10 |
| Mean confidence for variety 31 data | 0.46 |
| Majority ratio for variety 31 data | 0.50 |
| Classification results for variety 31 | The new data is classified as a new variety. |
| Mean confidence for variety 8094 data | 0.48 |
| Majority ratio for variety 8094 data | 0.50 |
| Classification results for variety 8094 | The new data is classified as a new variety. |
| Accuracy | 0.40 |
| F1 Score | 0.38 |
| Precision | 0.39 |
| Recall | 0.40 |

| Set name | DATA d |
| --- | --- |
| Subset name | DATA d1 |
| Mean confidence for variety 31 data | 0.76 |
| Majority ratio for variety 31 data | 0.80 |
| Classification results for variety 31 | The new data is classified as a new variety. |
| Mean confidence for variety 8094 data | 0.71 |
| Majority ratio for variety 8094 data | 0.60 |
| Classification results for variety 8094 | The new data is classified as a new variety. |
| Accuracy | 0.51 |
| F1 Score | 0.51 |
| Precision | 0.54 |
| Recall | 0.51 |

| Set name | DATA d |
| --- | --- |
| Subset name | DATA d2 |
| Mean confidence for variety 31 data | 0.50 |
| Majority ratio for variety 31 data | 0.40 |
| Classification results for variety 31 | The new data is classified as a new variety. |
| Mean confidence for variety 8094 data | 0.65 |
| Majority ratio for variety 8094 data | 0.50 |
| Classification results for variety 8094 | The new data is classified as a new variety. |
| Accuracy | 0.38 |
| F1 Score | 0.35 |
| Precision | 0.36 |
| Recall | 0.38 |

| Set name | DATA d |
| --- | --- |
| Subset name | DATA d3 |
| Mean confidence for variety 31 data | 0.72 |
| Majority ratio for variety 31 data | 1.00 |
| Classification results for variety 31 | The new data is classified as a new variety. |
| Mean confidence for variety 8094 data | 0.67 |
| Majority ratio for variety 8094 data | 0.40 |
| Classification results for variety 8094 | The new data is classified as a new variety. |
| Accuracy | 0.53 |
| F1 Score | 0.52 |
| Precision | 0.54 |
| Recall | 0.53 |

| Set name | DATA d |
| --- | --- |
| Subset name | DATA d4 |
| Mean confidence for variety 31 data | 0.60 |
| Majority ratio for variety 31 data | 0.60 |
| Classification results for variety 31 | The new data is classified as a new variety. |
| Mean confidence for variety 8094 data | 0.82 |
| Majority ratio for variety 8094 data | 0.60 |
| Classification results for variety 8094 | The new data is classified as a new variety. |
| Accuracy | 0.65 |
| F1 Score | 0.65 |
| Precision | 0.64 |
| Recall | 0.65 |

| Set name | DATA d |
| --- | --- |
| Subset name | DATA d5 |
| Mean confidence for variety 31 data | 0.45 |
| Majority ratio for variety 31 data | 0.50 |
| Classification results for variety 31 | The new data is classified as a new variety. |
| Mean confidence for variety 8094 data | 0.69 |
| Majority ratio for variety 8094 data | 0.70 |
| Classification results for variety 8094 | The new data is classified as a new variety. |
| Accuracy | 0.59 |
| F1 Score | 0.58 |
| Precision | 0.60 |
| Recall | 0.59 |

| Set name | DATA d |
| --- | --- |
| Subset name | DATA d6 |
| Mean confidence for variety 31 data | 0.63 |
| Majority ratio for variety 31 data | 0.40 |
| Classification results for variety 31 | The new data is classified as a new variety. |
| Mean confidence for variety 8094 data | 0.72 |
| Majority ratio for variety 8094 data | 0.50 |
| Classification results for variety 8094 | The new data is classified as a new variety. |
| Accuracy | 0.57 |
| F1 Score | 0.57 |
| Precision | 0.59 |
| Recall | 0.57 |

| Set name | DATA d |
| --- | --- |
| Subset name | DATA d7 |
| Mean confidence for variety 31 data | 0.83 |
| Majority ratio for variety 31 data | 1.00 |
| Classification results for variety 31 | The new data is classified as variety 31. |
| Mean confidence for variety 8094 data | 0.68 |
| Majority ratio for variety 8094 data | 0.60 |
| Classification results for variety 8094 | The new data is classified as a new variety. |
| Accuracy | 0.68 |
| F1 Score | 0.68 |
| Precision | 0.68 |
| Recall | 0.68 |

| Set name | DATA d |
| --- | --- |
| Subset name | DATA d8 |
| Mean confidence for variety 31 data | 0.55 |
| Majority ratio for variety 31 data | 0.50 |
| Classification results for variety 31 | The new data is classified as a new variety. |
| Mean confidence for variety 8094 data | 0.73 |
| Majority ratio for variety 8094 data | 0.50 |
| Classification results for variety 8094 | The new data is classified as a new variety. |
| Accuracy | 0.55 |
| F1 Score | 0.55 |
| Precision | 0.57 |
| Recall | 0.55 |

| Set name | DATA d |
| --- | --- |
| Subset name | DATA d9 |
| Mean confidence for variety 31 data | 0.79 |
| Majority ratio for variety 31 data | 0.60 |
| Classification results for variety 31 | The new data is classified as a new variety. |
| Mean confidence for variety 8094 data | 0.74 |
| Majority ratio for variety 8094 data | 0.50 |
| Classification results for variety 8094 | The new data is classified as a new variety. |
| Accuracy | 0.72 |
| F1 Score | 0.70 |
| Precision | 0.71 |
| Recall | 0.72 |

| Set name | DATA d |
| --- | --- |
| Subset name | DATA d10 |
| Mean confidence for variety 31 data | 0.76 |
| Majority ratio for variety 31 data | 0.70 |
| Classification results for variety 31 | The new data is classified as a new variety. |
| Mean confidence for variety 8094 data | 0.67 |
| Majority ratio for variety 8094 data | 0.40 |
| Classification results for variety 8094 | The new data is classified as a new variety. |
| Accuracy | 0.63 |
| F1 Score | 0.63 |
| Precision | 0.65 |
| Recall | 0.63 |

| Set name | DATA d |
| --- | --- |
| Subset name | DATA d11 |
| Mean confidence for variety 31 data | 0.53 |
| Majority ratio for variety 31 data | 0.60 |
| Classification results for variety 31 | The new data is classified as a new variety. |
| Mean confidence for variety 8094 data | 0.67 |
| Majority ratio for variety 8094 data | 0.40 |
| Classification results for variety 8094 | The new data is classified as a new variety. |
| Accuracy | 0.61 |
| F1 Score | 0.60 |
| Precision | 0.60 |
| Recall | 0.61 |

| Set name | DATA d |
| --- | --- |
| Subset name | DATA d12 |
| Mean confidence for variety 31 data | 0.52 |
| Majority ratio for variety 31 data | 0.50 |
| Classification results for variety 31 | The new data is classified as a new variety. |
| Mean confidence for variety 8094 data | 0.61 |
| Majority ratio for variety 8094 data | 0.50 |
| Classification results for variety 8094 | The new data is classified as a new variety. |
| Accuracy | 0.50 |
| F1 Score | 0.51 |
| Precision | 0.53 |
| Recall | 0.50 |

| Set name | DATA d |
| --- | --- |
| Subset name | DATA d13 |
| Mean confidence for variety 31 data | 0.70 |
| Majority ratio for variety 31 data | 0.70 |
| Classification results for variety 31 | The new data is classified as a new variety. |
| Mean confidence for variety 8094 data | 0.86 |
| Majority ratio for variety 8094 data | 0.80 |
| Classification results for variety 8094 | The new data is classified as a new variety. |
| Accuracy | 0.83 |
| F1 Score | 0.83 |
| Precision | 0.84 |
| Recall | 0.83 |

| Set name | DATA d |
| --- | --- |
| Subset name | DATA d14 |
| Mean confidence for variety 31 data | 0.56 |
| Majority ratio for variety 31 data | 0.60 |
| Classification results for variety 31 | The new data is classified as a new variety. |
| Mean confidence for variety 8094 data | 0.76 |
| Majority ratio for variety 8094 data | 0.50 |
| Classification results for variety 8094 | The new data is classified as a new variety. |
| Accuracy | 0.52 |
| F1 Score | 0.51 |
| Precision | 0.51 |
| Recall | 0.52 |

| Set name | DATA d |
| --- | --- |
| Subset name | DATA d15 |
| Mean confidence for variety 31 data | 0.60 |
| Majority ratio for variety 31 data | 0.90 |
| Classification results for variety 31 | The new data is classified as a new variety. |
| Mean confidence for variety 8094 data | 0.63 |
| Majority ratio for variety 8094 data | 0.40 |
| Classification results for variety 8094 | The new data is classified as a new variety. |
| Accuracy | 0.46 |
| F1 Score | 0.46 |
| Precision | 0.47 |
| Recall | 0.46 |

| Set name | DATA e |
| --- | --- |
| Subset name | DATA e1 |
| Mean confidence for variety 31 data | 1.00 |
| Majority ratio for variety 31 data | 1.00 |
| Classification results for variety 31 | The new data is classified as variety 93. |
| Mean confidence for variety 8094 data | 1.00 |
| Majority ratio for variety 8094 data | 1.00 |
| Classification results for variety 8094 | The new data is classified as variety 93. |
| Accuracy | 0.25 |
| F1 Score | 0.10 |
| Precision | 0.06 |
| Recall | 0.25 |

| Set name | DATA e |
| --- | --- |
| Subset name | DATA e2 |
| Mean confidence for variety 31 data | 1.00 |
| Majority ratio for variety 31 data | 1.00 |
| Classification results for variety 31 | The new data is classified as variety 103. |
| Mean confidence for variety 8094 data | 1.00 |
| Majority ratio for variety 8094 data | 1.00 |
| Classification results for variety 8094 | The new data is classified as variety 103. |
| Accuracy | 0.25 |
| F1 Score | 0.10 |
| Precision | 0.06 |
| Recall | 0.25 |

| Set name | DATA e |
| --- | --- |
| Subset name | DATA e3 |
| Mean confidence for variety 31 data | 0.96 |
| Majority ratio for variety 31 data | 1.00 |
| Classification results for variety 31 | The new data is classified as variety 73. |
| Mean confidence for variety 8094 data | 0.97 |
| Majority ratio for variety 8094 data | 1.00 |
| Classification results for variety 8094 | The new data is classified as variety 73. |
| Accuracy | 0.25 |
| F1 Score | 0.10 |
| Precision | 0.06 |
| Recall | 0.25 |

| Set name | DATA e |
| --- | --- |
| Subset name | DATA e4 |
| Mean confidence for variety 31 data | 1.00 |
| Majority ratio for variety 31 data | 1.00 |
| Classification results for variety 31 | The new data is classified as variety 114. |
| Mean confidence for variety 8094 data | 1.00 |
| Majority ratio for variety 8094 data | 1.00 |
| Classification results for variety 8094 | The new data is classified as variety 114. |
| Accuracy | 0.25 |
| F1 Score | 0.10 |
| Precision | 0.06 |
| Recall | 0.25 |

| Set name | DATA e |
| --- | --- |
| Subset name | DATA e5 |
| Mean confidence for variety 31 data | 0.98 |
| Majority ratio for variety 31 data | 1.00 |
| Classification results for variety 31 | The new data is classified as variety 92. |
| Mean confidence for variety 8094 data | 0.69 |
| Majority ratio for variety 8094 data | 0.80 |
| Classification results for variety 8094 | The new data is classified as a new variety. |
| Accuracy | 0.24 |
| F1 Score | 0.10 |
| Precision | 0.07 |
| Recall | 0.24 |

| Set name | DATA e |
| --- | --- |
| Subset name | DATA e6 |
| Mean confidence for variety 31 data | 0.90 |
| Majority ratio for variety 31 data | 1.00 |
| Classification results for variety 31 | The new data is classified as variety 75. |
| Mean confidence for variety 8094 data | 0.71 |
| Majority ratio for variety 8094 data | 0.60 |
| Classification results for variety 8094 | The new data is classified as a new variety. |
| Accuracy | 0.20 |
| F1 Score | 0.10 |
| Precision | 0.06 |
| Recall | 0.20 |

| Set name | DATA e |
| --- | --- |
| Subset name | DATA e7 |
| Mean confidence for variety 31 data | 1.00 |
| Majority ratio for variety 31 data | 1.00 |
| Classification results for variety 31 | The new data is classified as variety 33. |
| Mean confidence for variety 8094 data | 0.86 |
| Majority ratio for variety 8094 data | 0.60 |
| Classification results for variety 8094 | The new data is classified as a new variety. |
| Accuracy | 0.24 |
| F1 Score | 0.10 |
| Precision | 0.06 |
| Recall | 0.24 |

| Set name | DATA e |
| --- | --- |
| Subset name | DATA e8 |
| Mean confidence for variety 31 data | 1.00 |
| Majority ratio for variety 31 data | 1.00 |
| Classification results for variety 31 | The new data is classified as variety 20. |
| Mean confidence for variety 8094 data | 1.00 |
| Majority ratio for variety 8094 data | 1.00 |
| Classification results for variety 8094 | The new data is classified as variety 20. |
| Accuracy | 0.27 |
| F1 Score | 0.13 |
| Precision | 0.33 |
| Recall | 0.27 |

| Set name | DATA e |
| --- | --- |
| Subset name | DATA e9 |
| Mean confidence for variety 31 data | 1.00 |
| Majority ratio for variety 31 data | 1.00 |
| Classification results for variety 31 | The new data is classified as variety 49. |
| Mean confidence for variety 8094 data | 0.99 |
| Majority ratio for variety 8094 data | 1.00 |
| Classification results for variety 8094 | The new data is classified as variety 49. |
| Accuracy | 0.26 |
| F1 Score | 0.12 |
| Precision | 0.19 |
| Recall | 0.26 |

| Set name | DATA e |
| --- | --- |
| Subset name | DATA e10 |
| Mean confidence for variety 31 data | 1.00 |
| Majority ratio for variety 31 data | 1.00 |
| Classification results for variety 31 | The new data is classified as variety 106. |
| Mean confidence for variety 8094 data | 1.00 |
| Majority ratio for variety 8094 data | 1.00 |
| Classification results for variety 8094 | The new data is classified as variety 106. |
| Accuracy | 0.25 |
| F1 Score | 0.10 |
| Precision | 0.06 |
| Recall | 0.25 |

| Set name | DATA e |
| --- | --- |
| Subset name | DATA e11 |
| Mean confidence for variety 31 data | 1.00 |
| Majority ratio for variety 31 data | 1.00 |
| Classification results for variety 31 | The new data is classified as variety 110. |
| Mean confidence for variety 8094 data | 1.00 |
| Majority ratio for variety 8094 data | 1.00 |
| Classification results for variety 8094 | The new data is classified as variety 110. |
| Accuracy | 0.25 |
| F1 Score | 0.10 |
| Precision | 0.06 |
| Recall | 0.25 |

| Set name | DATA e |
| --- | --- |
| Subset name | DATA e12 |
| Mean confidence for variety 31 data | 1.00 |
| Majority ratio for variety 31 data | 1.00 |
| Classification results for variety 31 | The new data is classified as variety 31. |
| Mean confidence for variety 8094 data | 1.00 |
| Majority ratio for variety 8094 data | 1.00 |
| Classification results for variety 8094 | The new data is classified as variety 31. |
| Accuracy | 0.24 |
| F1 Score | 0.09 |
| Precision | 0.06 |
| Recall | 0.24 |

| Set name | DATA e |
| --- | --- |
| Subset name | DATA e13 |
| Mean confidence for variety 31 data | 0.82 |
| Majority ratio for variety 31 data | 1.00 |
| Classification results for variety 31 | The new data is classified as variety 10. |
| Mean confidence for variety 8094 data | 0.81 |
| Majority ratio for variety 8094 data | 0.90 |
| Classification results for variety 8094 | The new data is classified as variety 10. |
| Accuracy | 0.26 |
| F1 Score | 0.11 |
| Precision | 0.07 |
| Recall | 0.26 |

| Set name | DATA e |
| --- | --- |
| Subset name | DATA e14 |
| Mean confidence for variety 31 data | 1.00 |
| Majority ratio for variety 31 data | 1.00 |
| Classification results for variety 31 | The new data is classified as variety 8083. |
| Mean confidence for variety 8094 data | 0.99 |
| Majority ratio for variety 8094 data | 1.00 |
| Classification results for variety 8094 | The new data is classified as variety 8083. |
| Accuracy | 0.29 |
| F1 Score | 0.13 |
| Precision | 0.08 |
| Recall | 0.29 |

| Set name | DATA e |
| --- | --- |
| Subset name | DATA e15 |
| Mean confidence for variety 31 data | 1.00 |
| Majority ratio for variety 31 data | 1.00 |
| Classification results for variety 31 | The new data is classified as variety 28. |
| Mean confidence for variety 8094 data | 1.00 |
| Majority ratio for variety 8094 data | 1.00 |
| Classification results for variety 8094 | The new data is classified as variety 28. |
| Accuracy | 0.24 |
| F1 Score | 0.09 |
| Precision | 0.06 |
| Recall | 0.24 |

| Set name | DATA e |
| --- | --- |
| Subset name | DATA e16 |
| Mean confidence for variety 31 data | 1.00 |
| Majority ratio for variety 31 data | 1.00 |
| Classification results for variety 31 | The new data is classified as variety 82. |
| Mean confidence for variety 8094 data | 0.95 |
| Majority ratio for variety 8094 data | 0.80 |
| Classification results for variety 8094 | The new data is classified as a new variety. |
| Accuracy | 0.25 |
| F1 Score | 0.10 |
| Precision | 0.06 |
| Recall | 0.25 |

| Set name | DATA e |
| --- | --- |
| Subset name | DATA e17 |
| Mean confidence for variety 31 data | 1.00 |
| Majority ratio for variety 31 data | 1.00 |
| Classification results for variety 31 | The new data is classified as variety 80. |
| Mean confidence for variety 8094 data | 1.00 |
| Majority ratio for variety 8094 data | 1.00 |
| Classification results for variety 8094 | The new data is classified as variety 80. |
| Accuracy | 0.25 |
| F1 Score | 0.10 |
| Precision | 0.06 |
| Recall | 0.25 |

| Set name | DATA e |
| --- | --- |
| Subset name | DATA e18 |
| Mean confidence for variety 31 data | 0.70 |
| Majority ratio for variety 31 data | 1.00 |
| Classification results for variety 31 | The new data is classified as a new variety. |
| Mean confidence for variety 8094 data | 0.84 |
| Majority ratio for variety 8094 data | 0.90 |
| Classification results for variety 8094 | The new data is classified as variety 88. |
| Accuracy | 0.32 |
| F1 Score | 0.22 |
| Precision | 0.16 |
| Recall | 0.32 |

| Set name | DATA e |
| --- | --- |
| Subset name | DATA e19 |
| Mean confidence for variety 31 data | 1.00 |
| Majority ratio for variety 31 data | 1.00 |
| Classification results for variety 31 | The new data is classified as variety 69. |
| Mean confidence for variety 8094 data | 1.00 |
| Majority ratio for variety 8094 data | 1.00 |

| Classification results for variety 8094 | The new data is classified as variety 69. |
| --- | --- |
| Accuracy | 0.24 |
| F1 Score | 0.10 |
| Precision | 0.06 |
| Recall | 0.24 |

| Set name | DATA f* |
| --- | --- |
| Subset name | DATA f1 |
| Mean confidence for variety 31 data | 0.97 |
| Majority ratio for variety 31 data | 1.00 |
| Classification results for variety 31 | The new data is classified as variety 3. |
| Mean confidence for variety 8094 data | 0.89 |
| Majority ratio for variety 8094 data | 0.60 |
| Classification results for variety 8094 | The new data is classified as a new variety. |
| Accuracy | 0.87 |
| F1 Score | 0.87 |
| Precision | 0.88 |
| Recall | 0.87 |

*For DATA f only f1 subset is presented as the number of subsets is too large (38).

**Table 3. Plots for DATA d.**

| Subset name: DATA d1 |
| --- |
| 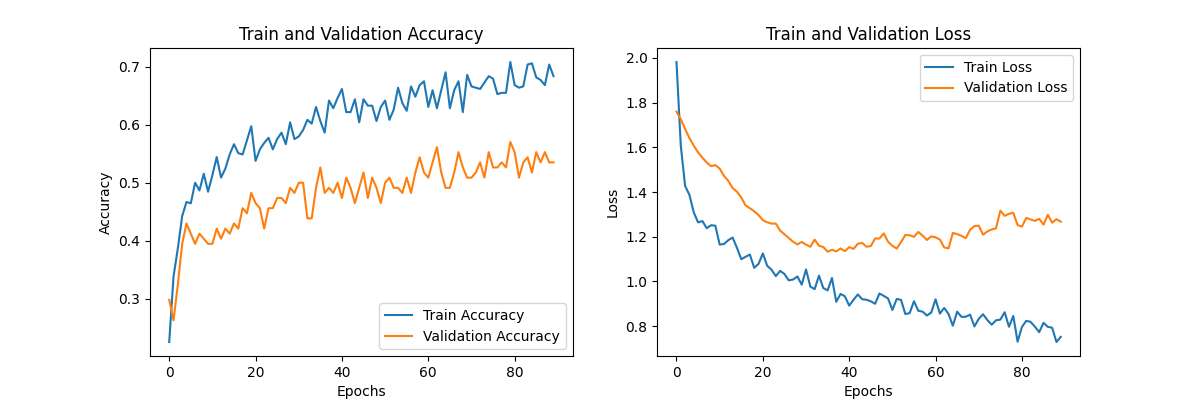 |
| 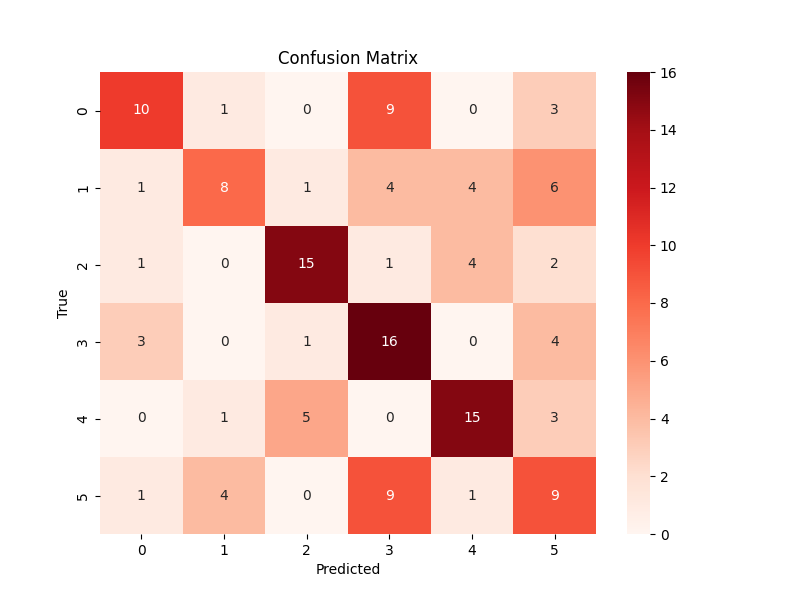 |

| Subset name: DATA d2 |
| --- |
| 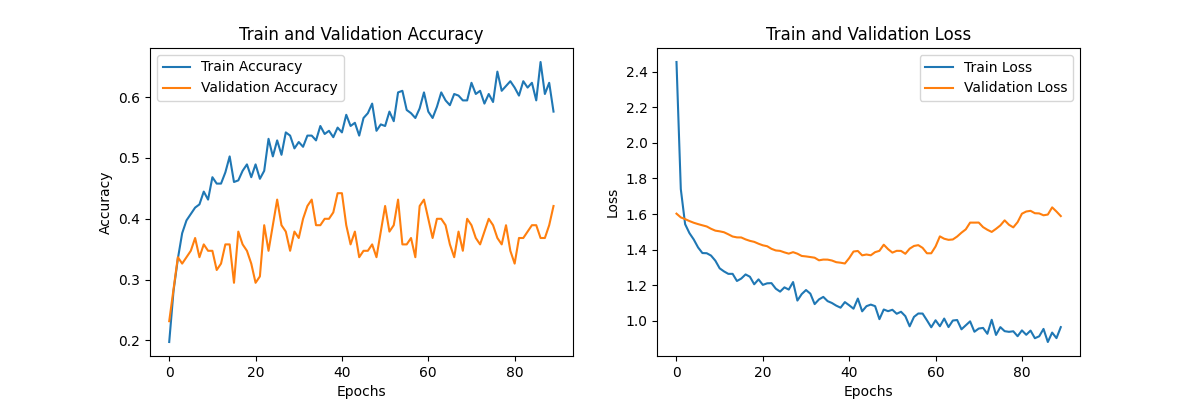 |
| 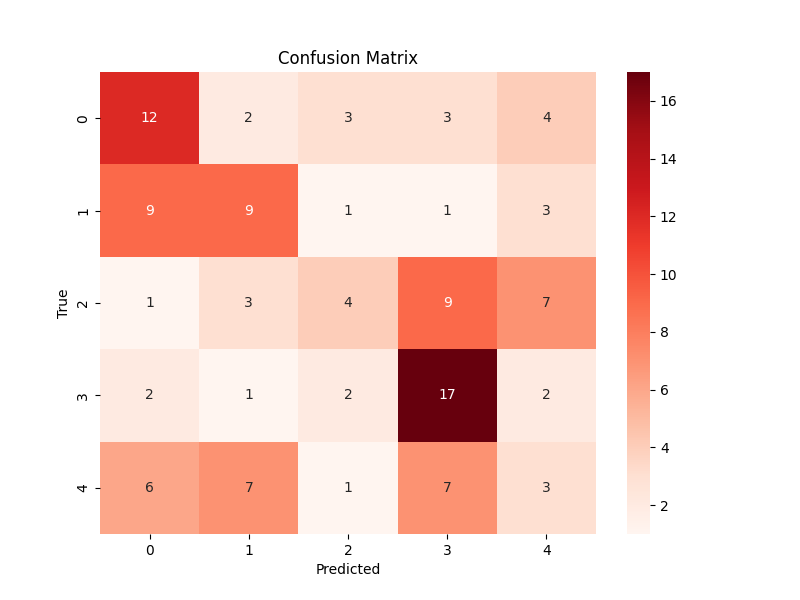 |

| Subset name: DATA d3 |
| --- |
| 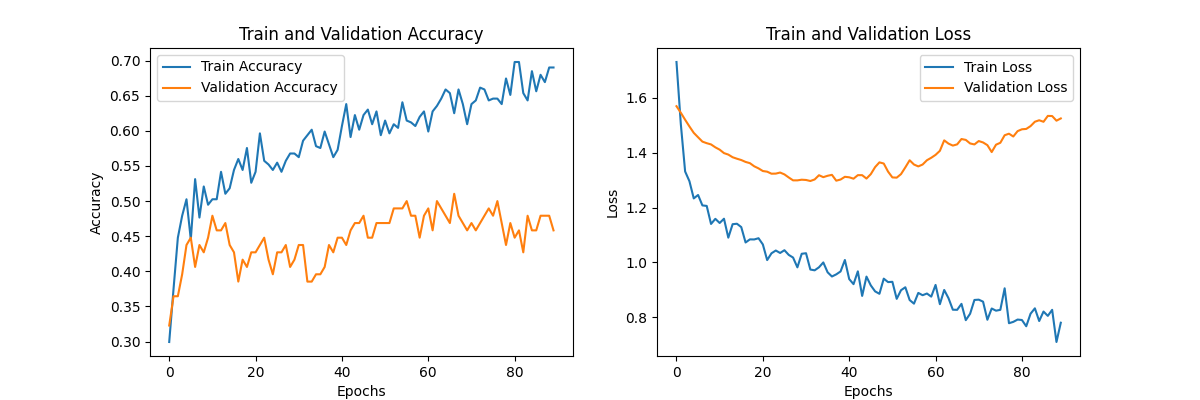 |
| 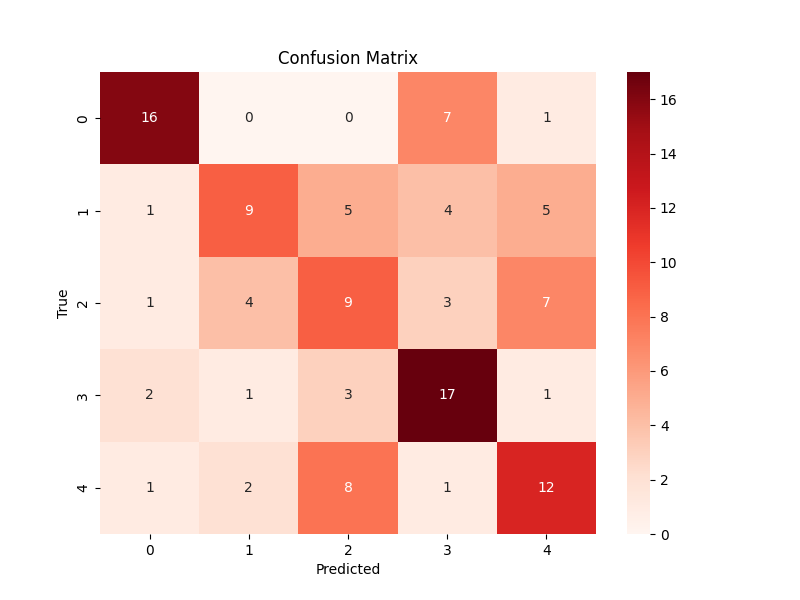 |

| Subset name: DATA d4 |
| --- |
| 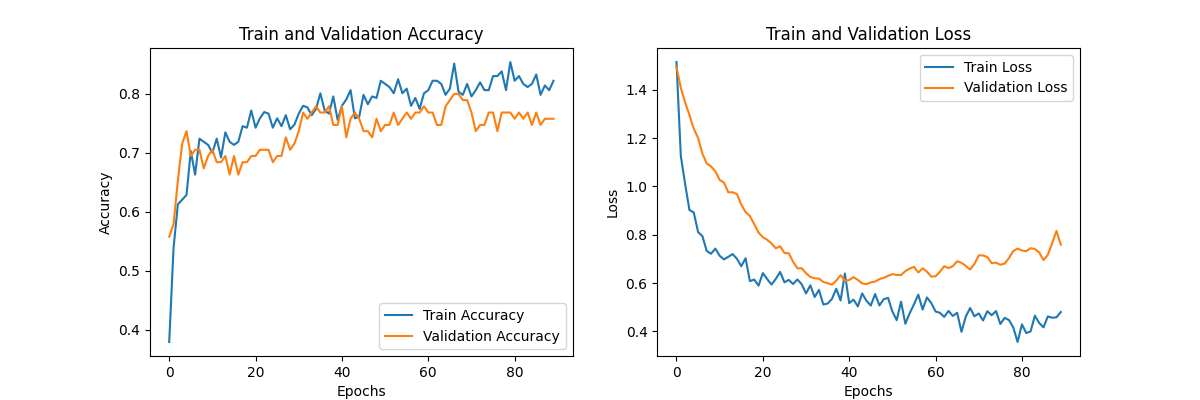 |
| 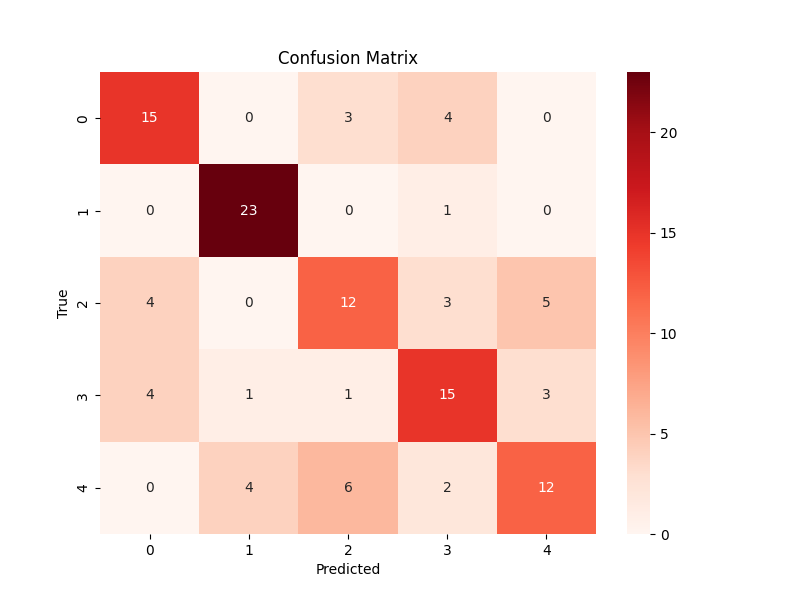 |

| Subset name: DATA d5 |
| --- |
| 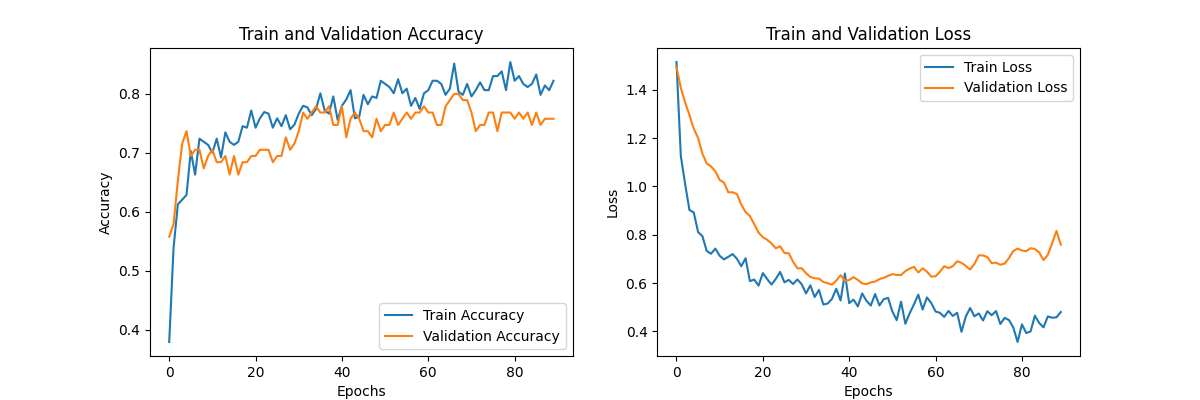 |
| 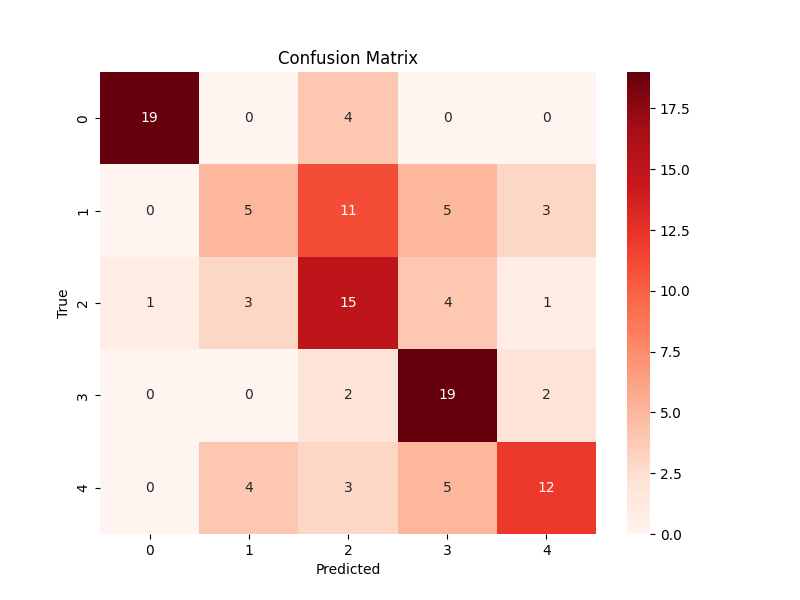 |

| Subset name: DATA d6 |
| --- |
| 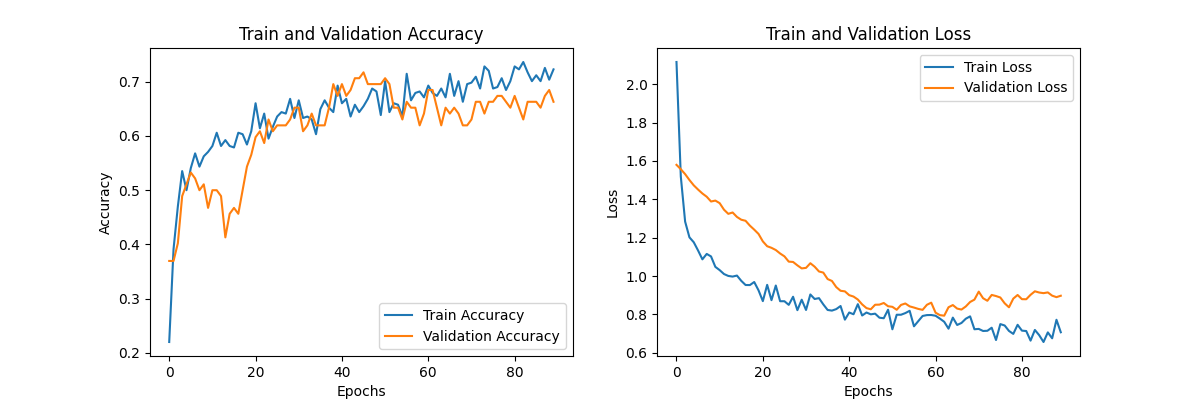 |
| 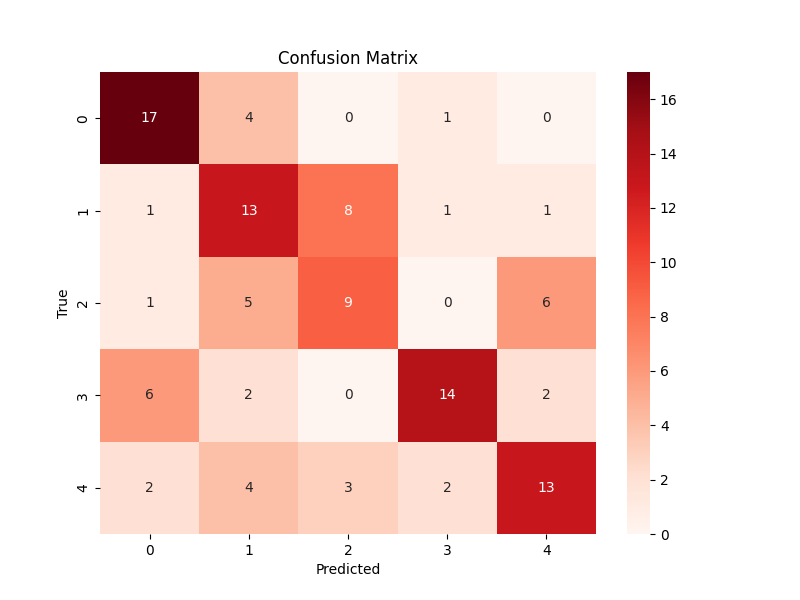 |

| Subset name: DATA d7 |
| --- |
| 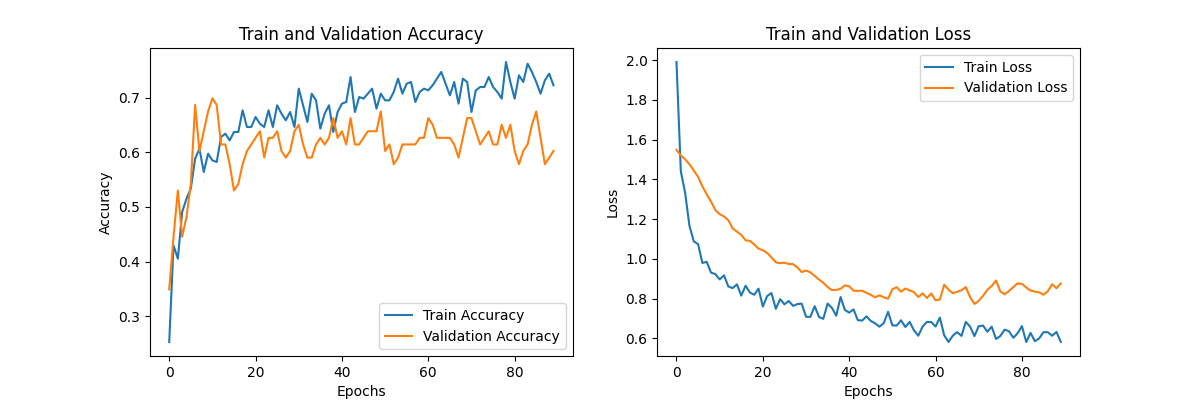 |
| 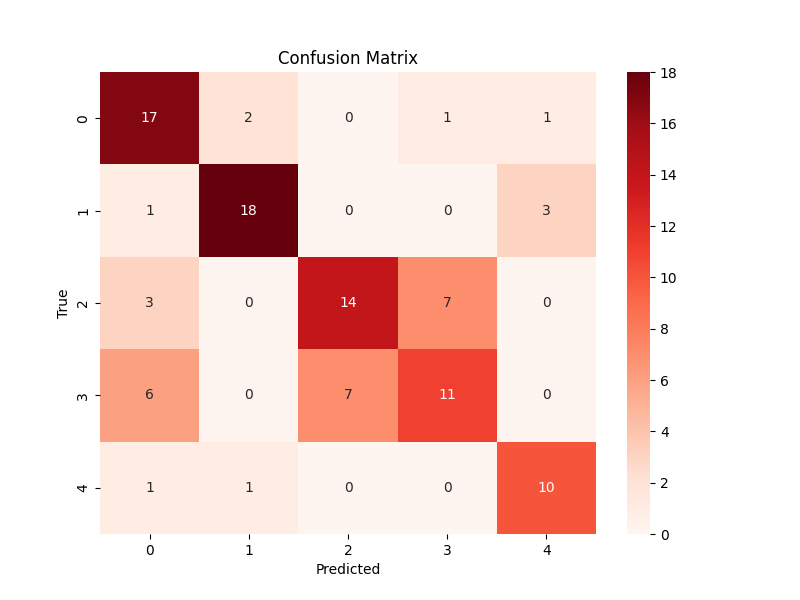 |

| Subset name: DATA d8 |
| --- |
| 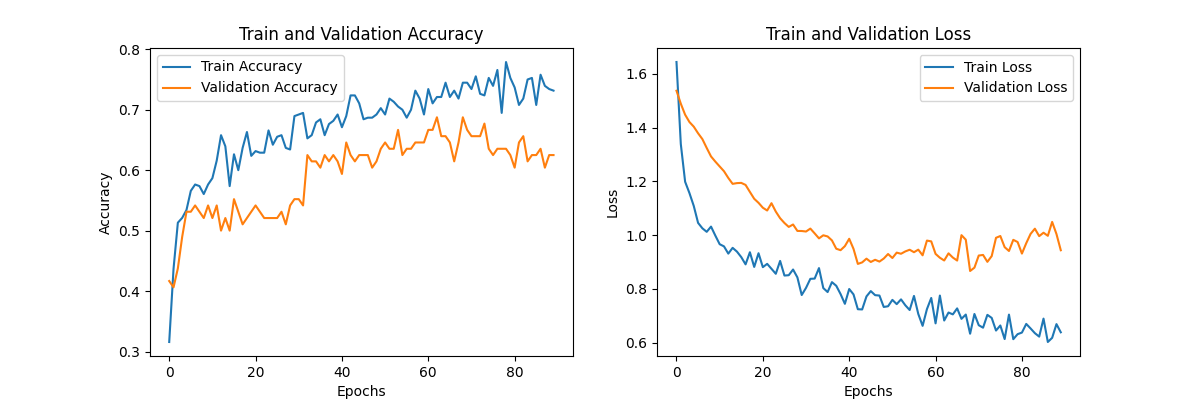 |
| 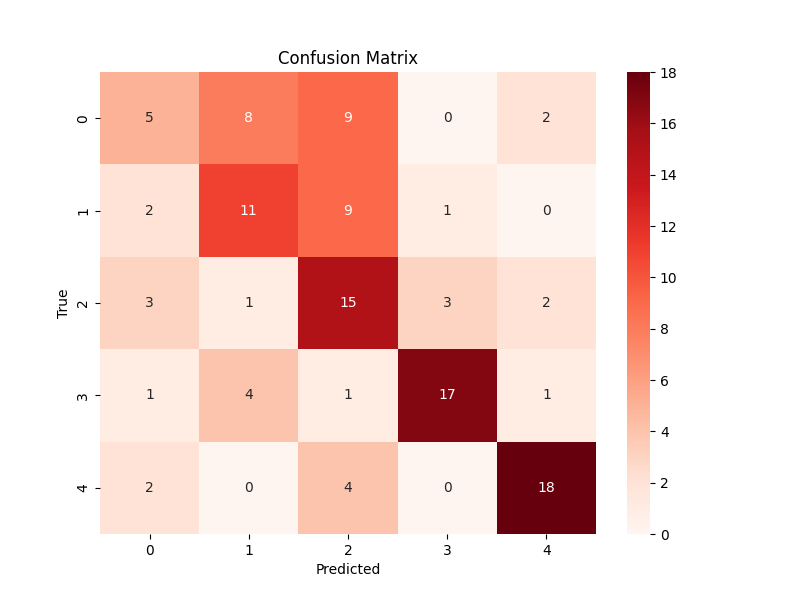 |

| Subset name: DATA d9 |
| --- |
| 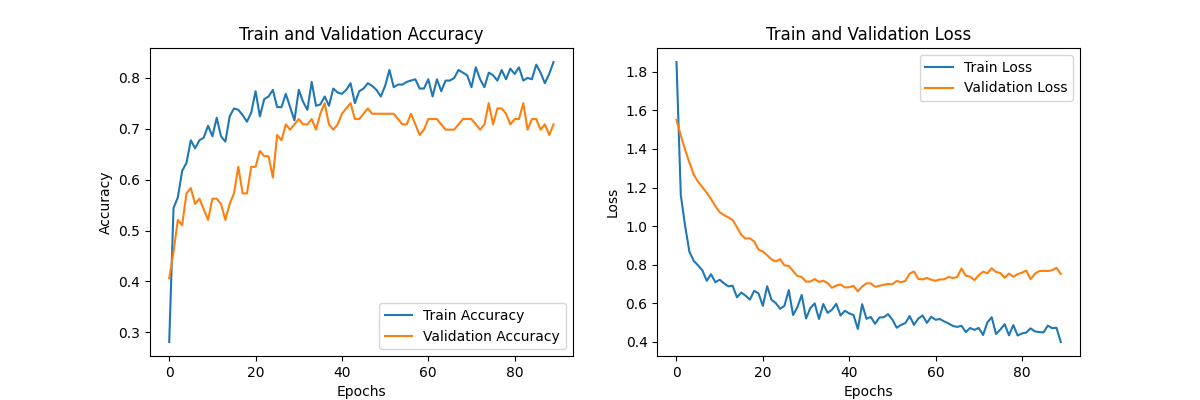 |
| 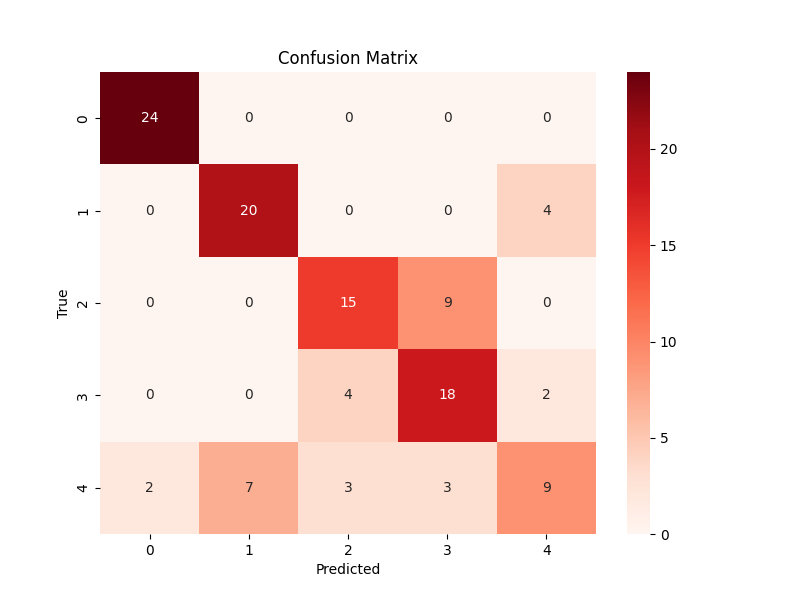 |

| Subset name: DATA d10 |
| --- |
| 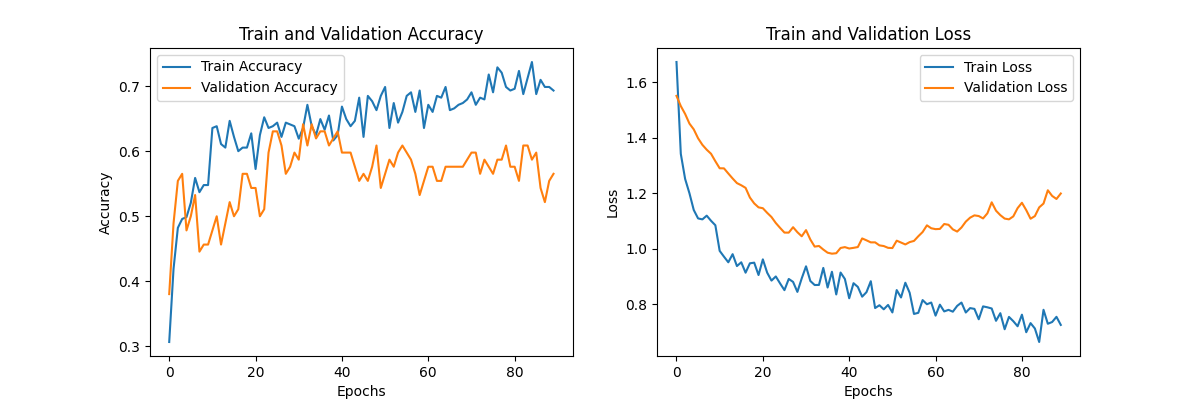 |
| 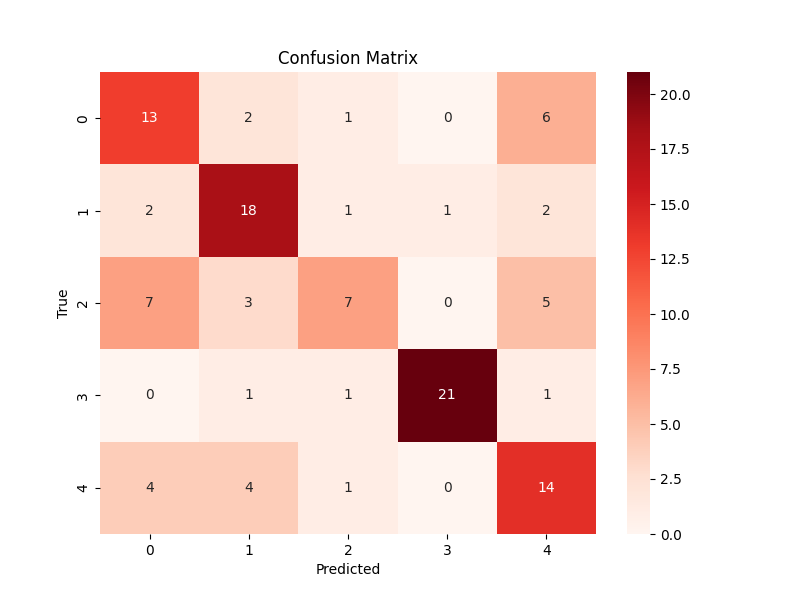 |

| Subset name: DATA d11 |
| --- |
| 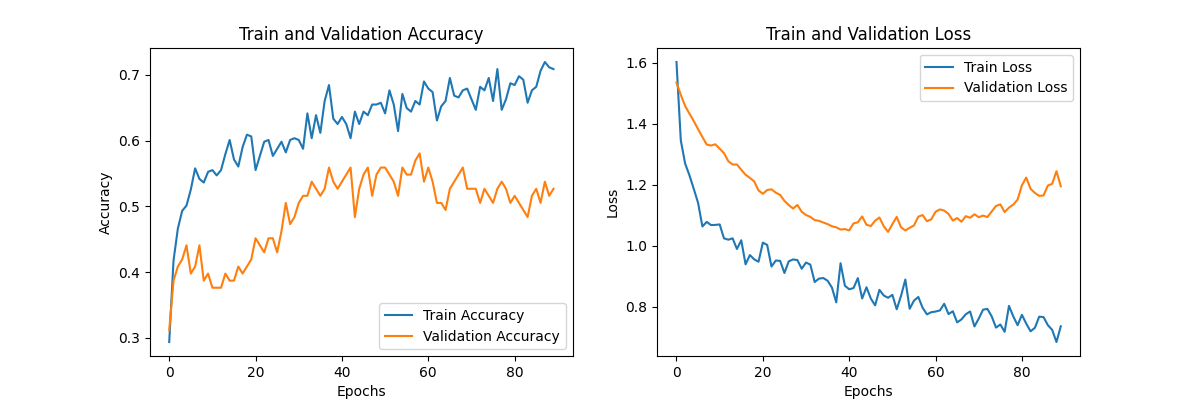 |
| 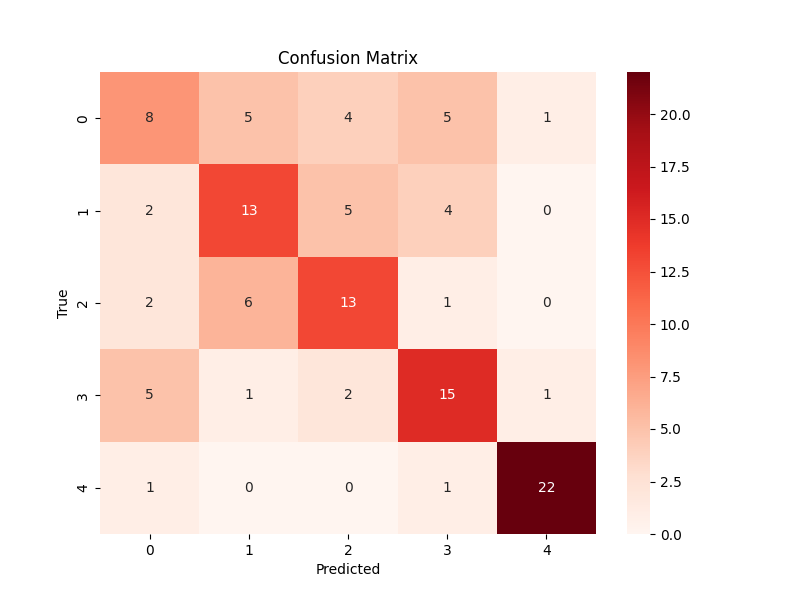 |

| Subset name: DATA d12 |
| --- |
| 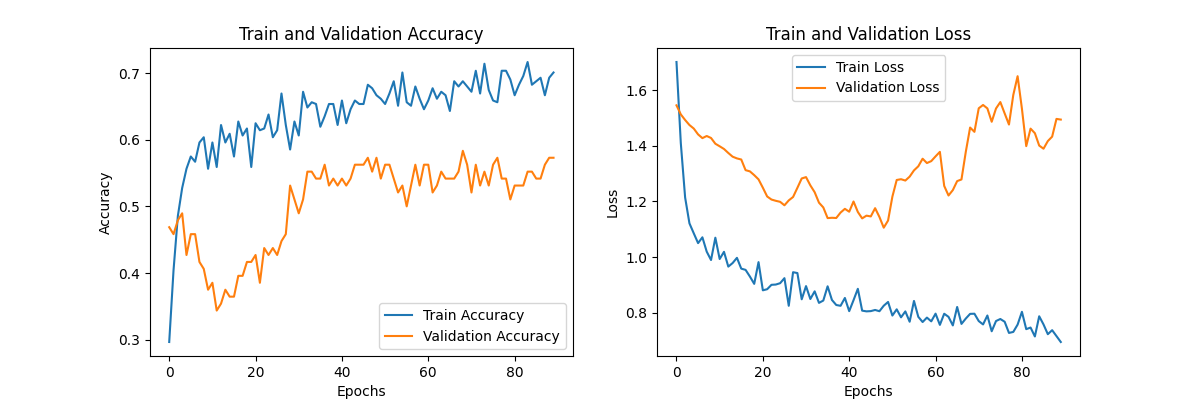 |
| 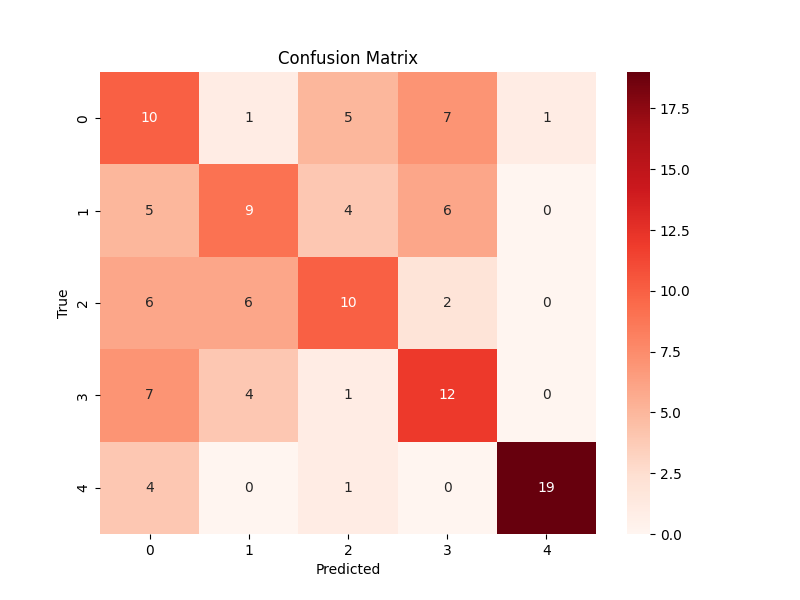 |

| Subset name: DATA d13 |
| --- |
| 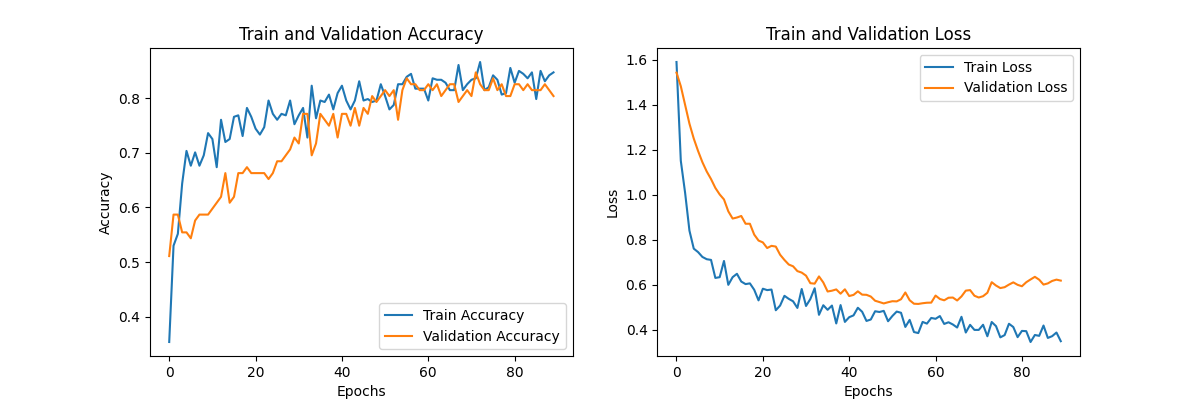 |
| 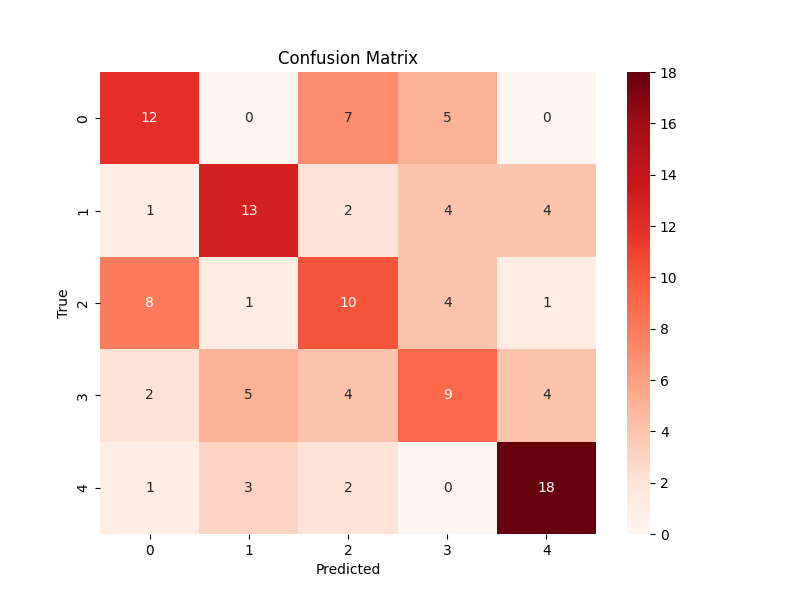 |

| Subset name: DATA d14 |
| --- |
| 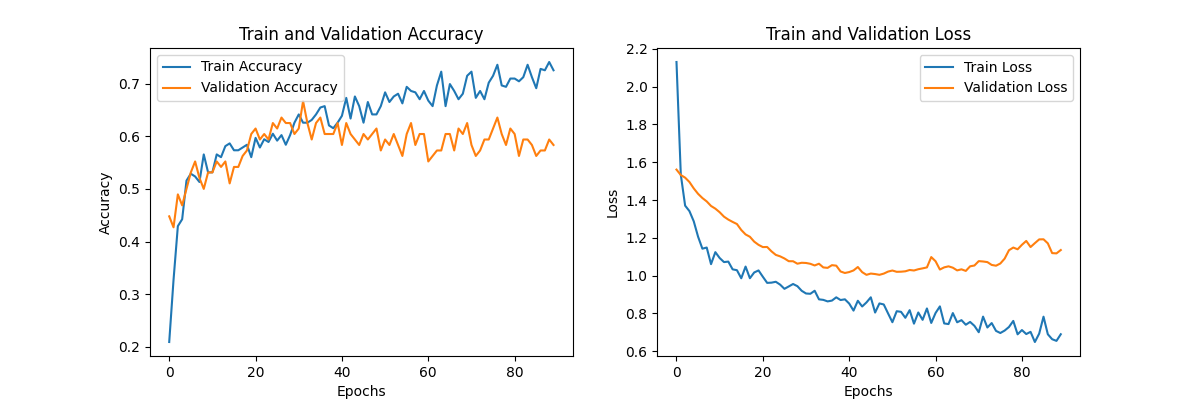 |
| 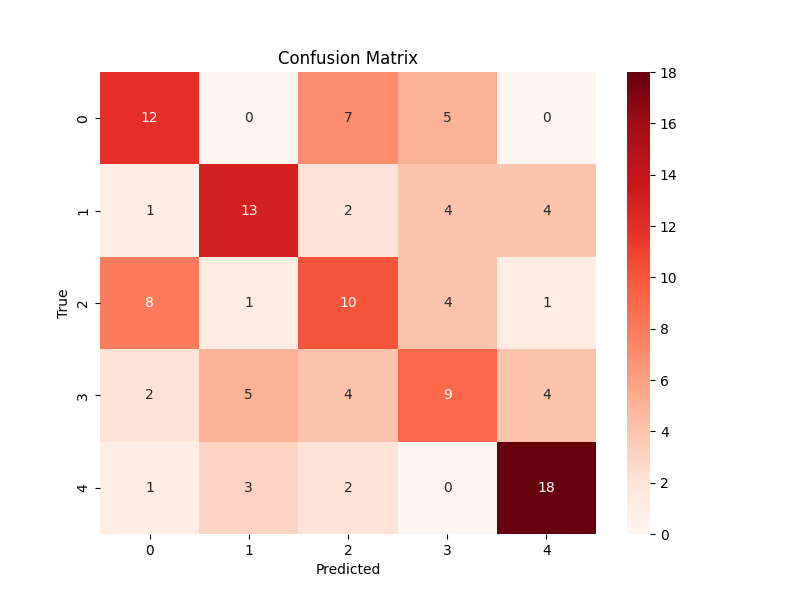 |

| Subset name: DATA d15 |
| --- |
| 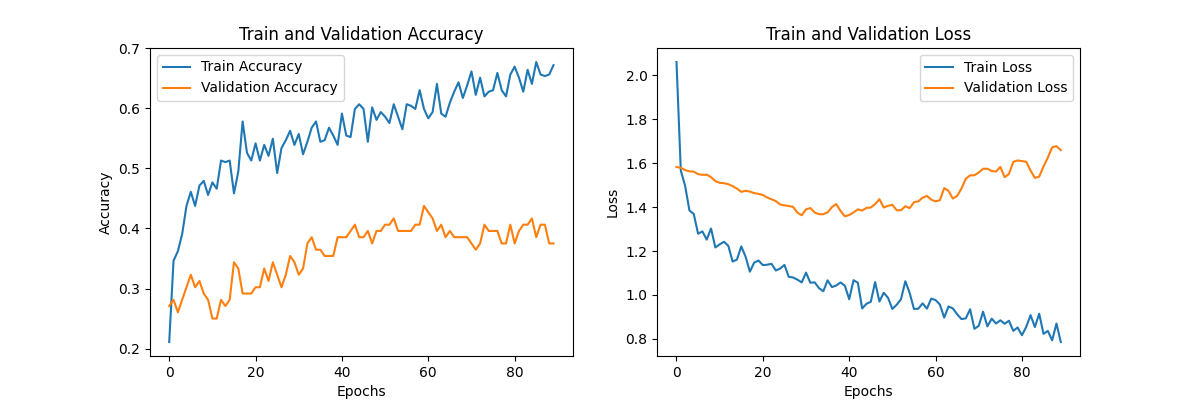 |
| 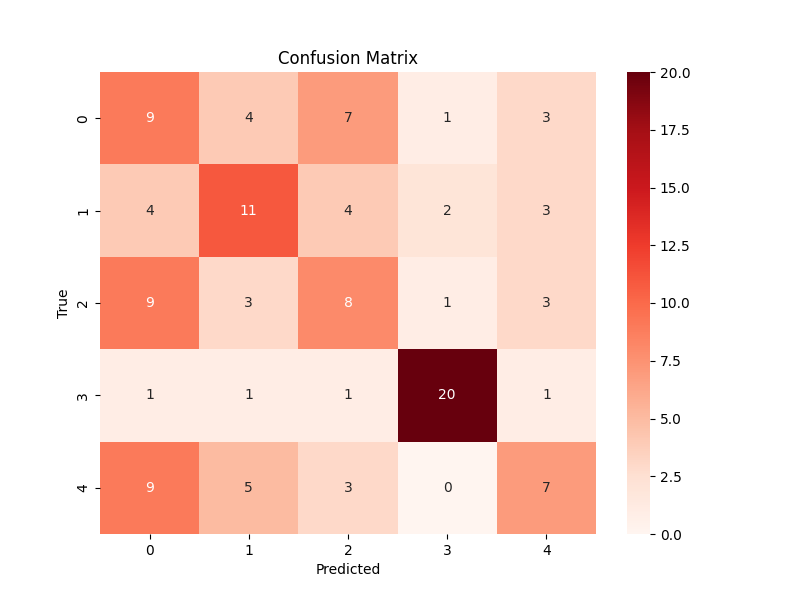 |
